# Ubiquitin ligase MARCH5 localizes to peroxisomes to regulate pexophagy

**DOI:** 10.1083/jcb.202103156

**Published:** 2021-11-08

**Authors:** Jun Zheng, Xi Chen, Qiang Liu, Guisheng Zhong, Min Zhuang

**Affiliations:** 1 School of Life Science and Technology, ShanghaiTech University, Shanghai, China; 2 Shanghai Institute of Biochemistry and Cell Biology, Center for Excellence in Molecular Cell Science, Chinese Academy of Sciences, Shanghai, China; 3 University of Chinese Academy of Sciences, Beijing, China; 4 Key Laboratory of Women’s Reproductive Health Research of Zhejiang Province, Women’s Hospital, Zhejiang University School of Medicine, Hangzhou City, Zhejiang Province, China; 5 iHuman Institute, ShanghaiTech University, Shanghai, China

## Abstract

Mitochondria and peroxisomes are independent but functionally closely related organelles. A few proteins have been characterized as dual-organelle locating proteins with distinct or similar roles on mitochondria and peroxisomes. MARCH5 is a mitochondria-associated ubiquitin ligase best known for its regulatory role in mitochondria quality control, fission, and fusion. Here, we used a proximity tagging system, PUP-IT, and identified new interacting proteins of MARCH5. Our data uncover that MARCH5 is a dual-organelle locating protein that interacts with several peroxisomal proteins. PEX19 binds the transmembrane region on MARCH5 and targets it to peroxisomes. On peroxisomes, MARCH5 binds and mediates the ubiquitination of PMP70. Furthermore, we find PMP70 ubiquitination and pexophagy induced by mTOR inhibition are blocked in the absence of MARCH5. Our study suggests novel roles of MARCH5 on peroxisomes.

## Teaser

Zheng et al. report that mitochondrial ubiquitin ligase MARCH5 is a dual-organelle locating protein that interacts with several peroxisomal proteins. Peroxisomal MARCH5 is required for mTOR inhibition–induced pexophagy by binding and ubiquitinating PMP70.

## Introduction

Peroxisomes are essential metabolic organelles that play critical roles in the metabolism of lipid and reactive oxygen species (ROS). They are compartments for fatty acid oxidation and ether lipid and bile acid synthesis. Peroxisomes generate ROS as by-products of the oxidative reactions and eliminate ROS with robust enzymes, such as H_2_O_2_-decomposing catalase. They also act as signaling platforms in innate immune signaling (Dixit et al., 2010; Odendall et al., 2014). Mammalian cells maintain peroxisome homeostasis with a balance between regulated biogenesis and degradation. The major peroxisome degradation pathway in mammalian cells is selected autophagy, also named pexophagy. Pexophagy is usually induced by upstream signals as a response to peroxisome quality control or metabolic stress. Despite the importance of maintaining peroxisome homeostasis, the molecular mechanisms of pexophagy in mammalian cells are not well established.

MARCH5 (also referred to as MITOL) is known as a mitochondrial ubiquitin ligase. It regulates the mitochondria homeostasis by ubiquitinating several important mitochondrial fusion/fission regulators (Karbowski et al., 2007; Park et al., 2014; Sugiura et al., 2013; Xu et al., 2016). MARCH5 generates the basal level of ubiquitination on mitochondria surface to recruit Parkin to mediate mitophagy (Koyano et al., 2019b). It also functions as one autophagy sensor to degrade FUNDC1 in response to hypoxia (Chen et al., 2017a, 2017b). Biochemical studies have revealed many proteins as the substrates of ubiquitin ligase MARCH5. MARCH5 targets mitofusins (Mfn1 and Mfn2) for ubiquitination, thus fine-tuning Mfn1 levels and regulating mitochondria and ER contacting sites (Park et al., 2014; Sugiura et al., 2013). It also targets DNM1L and MiD49, both necessary for mitochondria fission (Karbowski et al., 2007). Other characterized MARCH5 substrates include the antiviral signaling protein MAVS and the pathogenic hepatitis B viral protein HBX (Yoo et al., 2015, 2019). More recently, MARCH5 was found to be associated with mitochondrial translocase to oppose protein import (Phu et al., 2020). The diverse substrates and broad spectrum of MARCH5-regulated pathways suggest the important role of MARCH5 in maintaining organelle function.

In this study, we used a proximity labeling method, PUP-IT (pupylation-based interaction tagging), to systematically identify potential MARCH5-interacting proteins. Surprisingly, we found that MARCH5 interacts with several peroxisome proteins, including PEX19, PEX3, and peroxisome membrane protein (PMP) 70. We characterized MARCH5 as a dual-organelle locating protein that can be targeted to peroxisomes by PEX19/PEX3. More strikingly, we found that MARCH5 mediates the ubiquitination of PMP70 and plays a role in mammalian target of rapamycin (mTOR) inhibition–induced pexophagy.

## Results

### Identification of MARCH5-interacting proteins with proximity tagging

**Figure 1.**
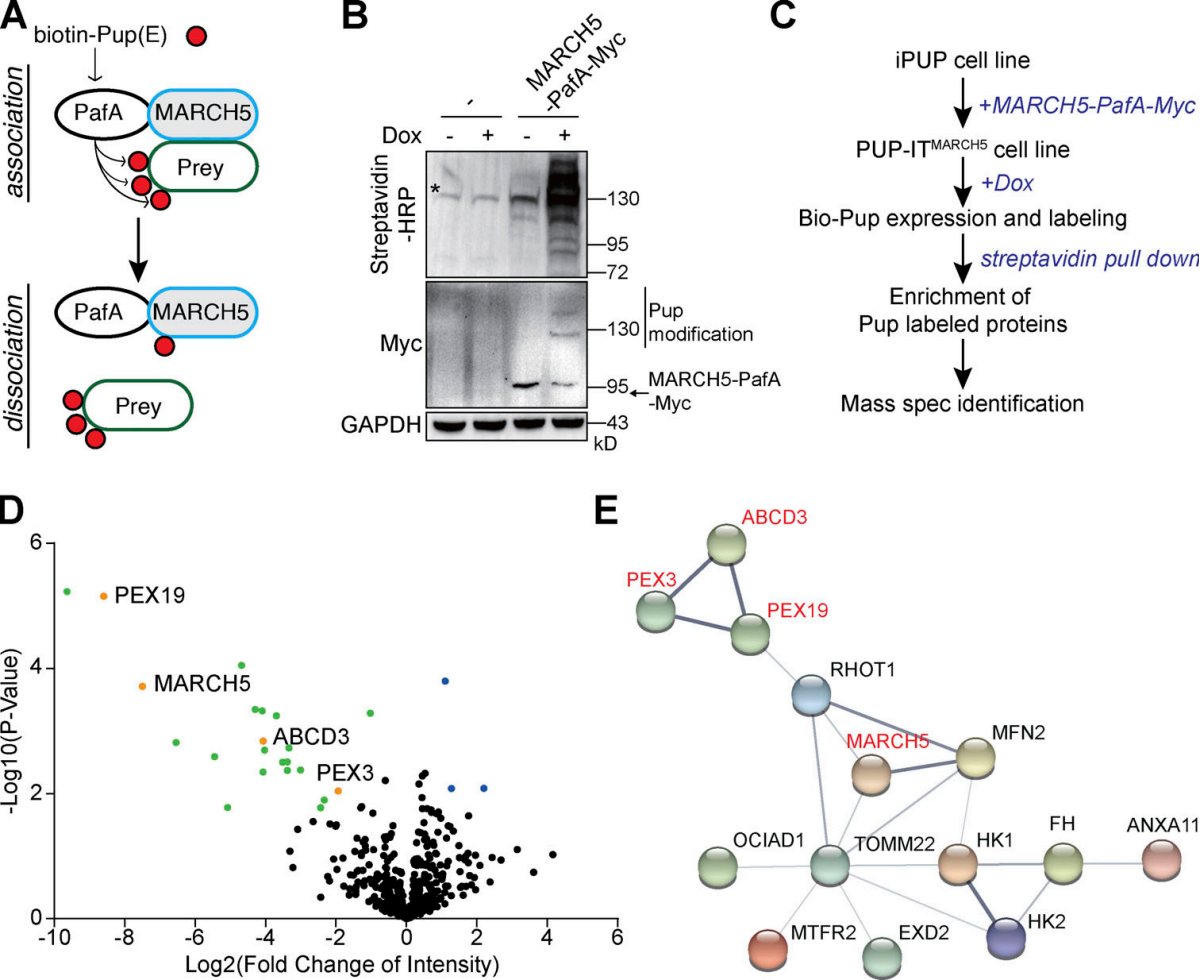
**Identification of MARCH5-interacting proteins by PUP-IT. (A)** Design of PUP-IT^MARCH5^. Pup(E) ligase PafA was fused to the C-terminus of MARCH5 and catalyzed biotin-Pup(E) ligation to MARCH5-interacting proteins. **(B)** iPUP cells expressing MARCH5-PafA-Myc were treated with Dox for 24 h. iPUP cells without MARCH5-PafA-Myc were used as the control. Biotin-modified proteins were analyzed with streptavidin-HRP. The asterisk indicates background from endogenous biotin-modified proteins. **(C)** The workflow of PUP-IT^MARCH5^–based proximity labeling to identify MARCH5-interacting proteins. **(D)** Volcano plot of proteins enriched as MARCH5-interacting proteins. The logarithmic ratios of protein LFQ intensity (iPUP/PUP-IT^MARCH5^) were plotted against negative logarithmic P values from a two-sided, two-sample *t* test in Perseus. Green and orange dots represent proteins that are enriched in the PUP-IT^MARCH5^ sample (FDR ≤0.05; *n* = 3 independent experiments). **(E)** Proteins enriched in PUP-IT^MARCH5^ sample were subjected to gene ontology analysis. STRING (functional protein-association networks) was used to analyze protein interactions (https://string-db.org/). The thickness of the lines indicates the strength of data support. Spec, spectrometry.

To further understand the molecular function of MARCH5, we profiled MARCH5-interacting proteins with the proximity tagging system PUP-IT (Liu et al., 2018). Bacteria-derived Pup ligase PafA is genetically fused to the C-terminus of MARCH5 with a linker of Gly/Ser. In the presence of Pup(E), PafA activates Pup(E) carboxyl glutamate in an ATP-dependent manner and catalyzes the covalent linkage between the glutamate and the side chain of a lysine on proximal proteins. This approach generates a stable marker on proteins and allows us to identify MARCH5-interacting proteins independent of the association or dissociation status between MARCH5 and the interacting proteins during sample preparation (Fig. 1 A).

To generate the iPUP cell line, we fused the biotin containing biotin carboxyl carrier protein domain at the N-terminus of Pup(E) to generate biotin-Pup(E), which is stably incorporated in Jurkat cells under a promotor controlled by the Tet-On system. Therefore, proximity labeling can be initiated by the addition of Tet-On inducer doxycycline (Dox). The efficiency of the proximity labeling can be measured by the biotin signal on proteins. We first tested whether Myc-tagged MARCH5-PafA (MARCH5-PafA-Myc) can mediate proximity labeling in iPUP cells. Only in the presence of MARCH5-PafA-Myc and Dox could we observe strong biotin signals in cell lysate (Fig. 1 B). Under the same condition, MARCH5-PafA-Myc self-modification could also be observed, showing multiple bands with larger molecular weight on Western blots (WBs; Fig. 1 B).

**Figure S1.**
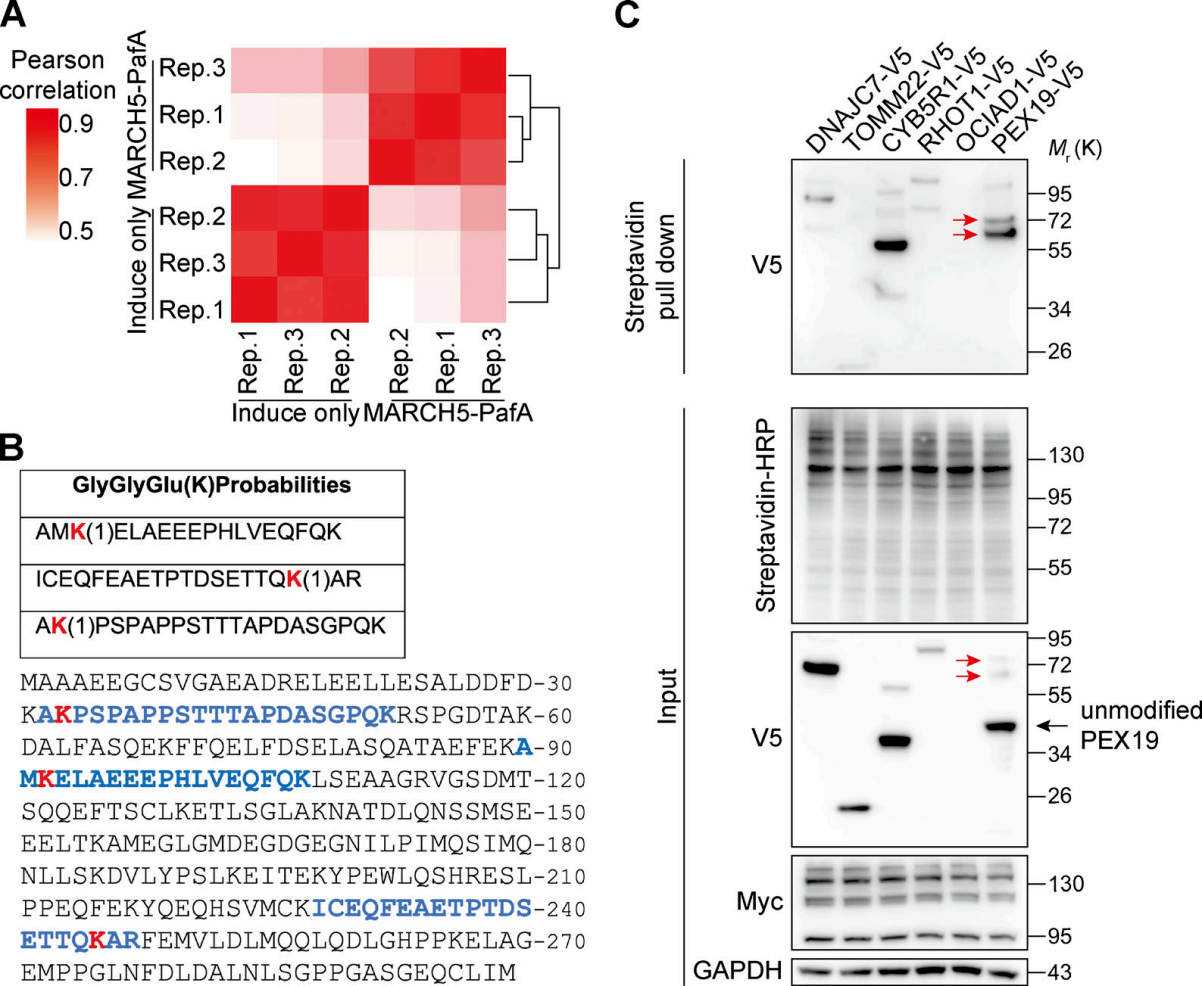
**Identification and validation of PEX19 as a MARCH5-interacting protein. (A)** Reproducibility of the proximity labeling experiments to identify MARCH5-interacting proteins. Pearson’s correlation coefficients between replicate (Rep.) MS results for PUP-IT^MARCH5^ and control samples. **(B)** PEX19 peptides containing GGE modification on lysine residues identified by MS. Red highlights the modification sites. Blue highlights the peptides in the context of full-length PEX19. **(C)** Multiple MARCH5-interacting candidates, including PEX19, are modified with biotin-Pup, showing higher-molecular-weight bands on WBs. HeLa cells were cotransfected with MARCH5-PafA-Myc and V5-tagged interacting candidate proteins. Proteins labeled by biotin-Pup were enriched by streptavidin magnetic beads, then analyzed by WB with anti-V5 antibodies. Black arrow, unmodified PEX19-V5 (expected, 35 kD); red arrows, biotin-Pup–modified PEX19-V5 (expected, 55 kD for monomodification and 75 kD for dimodification).

To identify biotin-Pup(E)–modified proteins in the presence of MARCH5-PafA-Myc, we followed the procedure in Fig. 1 C by coupling proximity labeling with mass spectrometry (MS). The proximity labeling and identification of MARCH5-interacting proteins were performed in three biological repeats and showed great reproducibility (Fig. S1 A). We identified 33 MARCH5-specific interacting proteins by spectral counts and 21 proteins by label-free quantification (LFQ) intensity (Fig. 1 D and Table S1). Among those proteins, most were mitochondrial proteins forming a highly connected protein–protein interaction network (Fig. 1 E), consistent with the major role of MARCH5 on mitochondria. Known MARCH5 substrate MFN2 has also been identified, validating the efficiency of proximity labeling. Surprisingly, three peroxisome-related proteins, PEX19, PEX3, and ABCD3, stood out as potential MARCH5-interacting proteins. All three proteins were identified with high confidence, showing a large fold change of LFQ intensity and more spectral counts than control samples (Fig. 1 D and Table S1). MARCH5-PafA–mediated PEX19 modification was also confirmed. Three PEX19 peptides modified by Pup(E) were identified with MS (Fig. S1 B). Bands with larger molecular weight, indicating posttranslational modifications with Pup(E), were detected on PEX19 WBs (Fig. S1 C).

### MARCH5 interacts with PEX19 and PEX3

PEX19 and PEX3 cooperate to assist PMP integration. Cytosolic PEX19 binds to the transmembrane (TM) region of cytosolic-translated PMPs, while membrane-anchored PEX3 functions as a PEX19 docking site on the peroxisome surface (Fang et al., 2004; Jansen and van der Klei, 2019; Schmidt et al., 2010). Although MARCH5 is a TM ubiquitin ligase mainly localized on mitochondria, we wondered whether it also localizes on peroxisomes via the same pathway as other PMPs.

**Figure 2.**
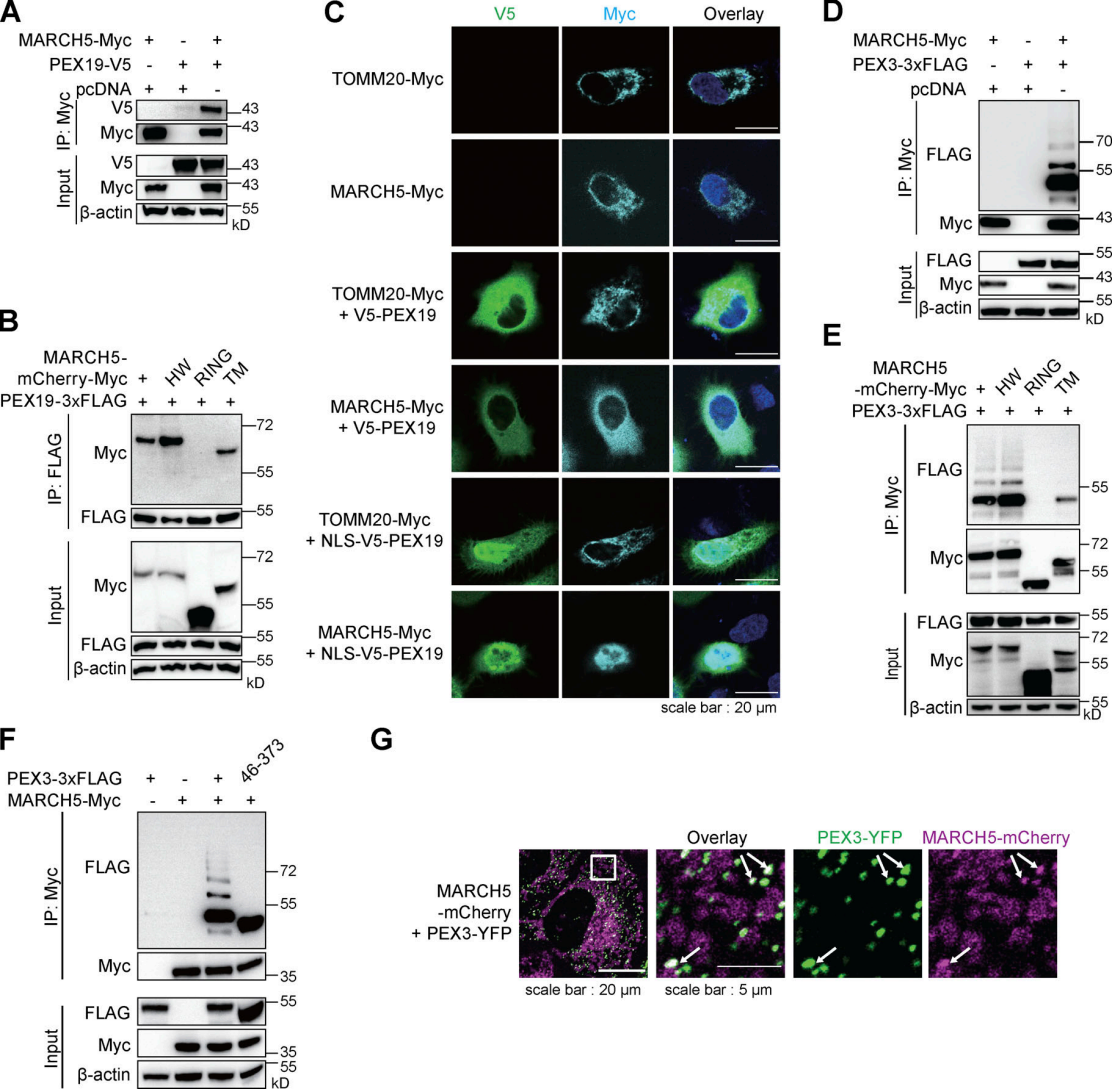
**MARCH5 interacts with PEX19 and PEX3. (A)** IP of MARCH5-Myc performed with HEK293T cells cotransfected with MARCH5-Myc and PEX19-V5. The samples were analyzed by IB with indicated antibodies. **(B)** HEK293T cells were cotransfected with PEX19-3×FLAG and different MARCH5-mCherry-Myc variants. Cells were collected 36 h after transfection for IP of PEX19-3×FLAG and IB with indicated antibodies. **(C)** V5-PEX19 or NLS fused V5-PEX19 were coexpressed with MARCH5-Myc or TOMM20-Myc in HeLa cells for 24 h. Cells are stained for Myc and V5 tags. **(D)** HEK293T cells were transfected with MARCH5-Myc and PEX3-3×FLAG for 30 h. Cells were treated with 50 μM MG132 for 6 h and then harvested for IP and IB with indicated antibodies. **(E)** HEK293T cells were cotransfected with PEX3-3×FLAG and different MARCH5-mCherry-Myc variants. IP and IB were performed with indicated antibodies. **(F)** HEK293T cells were transfected with MARCH5-Myc and PEX3-3×FLAG or PEX3(46–373)-3×FLAG for 36 h. Cells were treated with 50 μM MG132 for 6 h and then harvested for IP of MARCH5-Myc and IB with indicated antibodies. **(G)** Confocal fluorescent images of HeLa cells infected with lentiviruses expressing MARCH5-mCherry and PEX3-YFP. White arrows indicate where mCherry and YFP signals overlap.

We first validated the interaction between MARCH5 and PEX19. With both proteins overexpressed in cells, V5-tagged PEX19 coimmunoprecipitated with Myc-tagged MARCH5 (Fig. 2 A). MARCH5 contains a characteristic ubiquitin ligase RING domain at the N-terminus and four TM α-helices at the C-terminus. We further mapped the region that interacts with PEX19. Two MARCH5 truncations containing either the RING domain (residues 1–68) or the TM region (residues 70–279) were examined. The RING domain was dispensable for PEX19 interaction. H43W mutation (HW) on the MARCH5 RING domain that disrupts ubiquitin ligase activity did not affect PEX19 interaction (Fig. 2 B). These results suggest that PEX19 binds to the TM region on MARCH5, consistent with known PEX19 function for binding to the TM region on PMPs.

**Figure S2.**
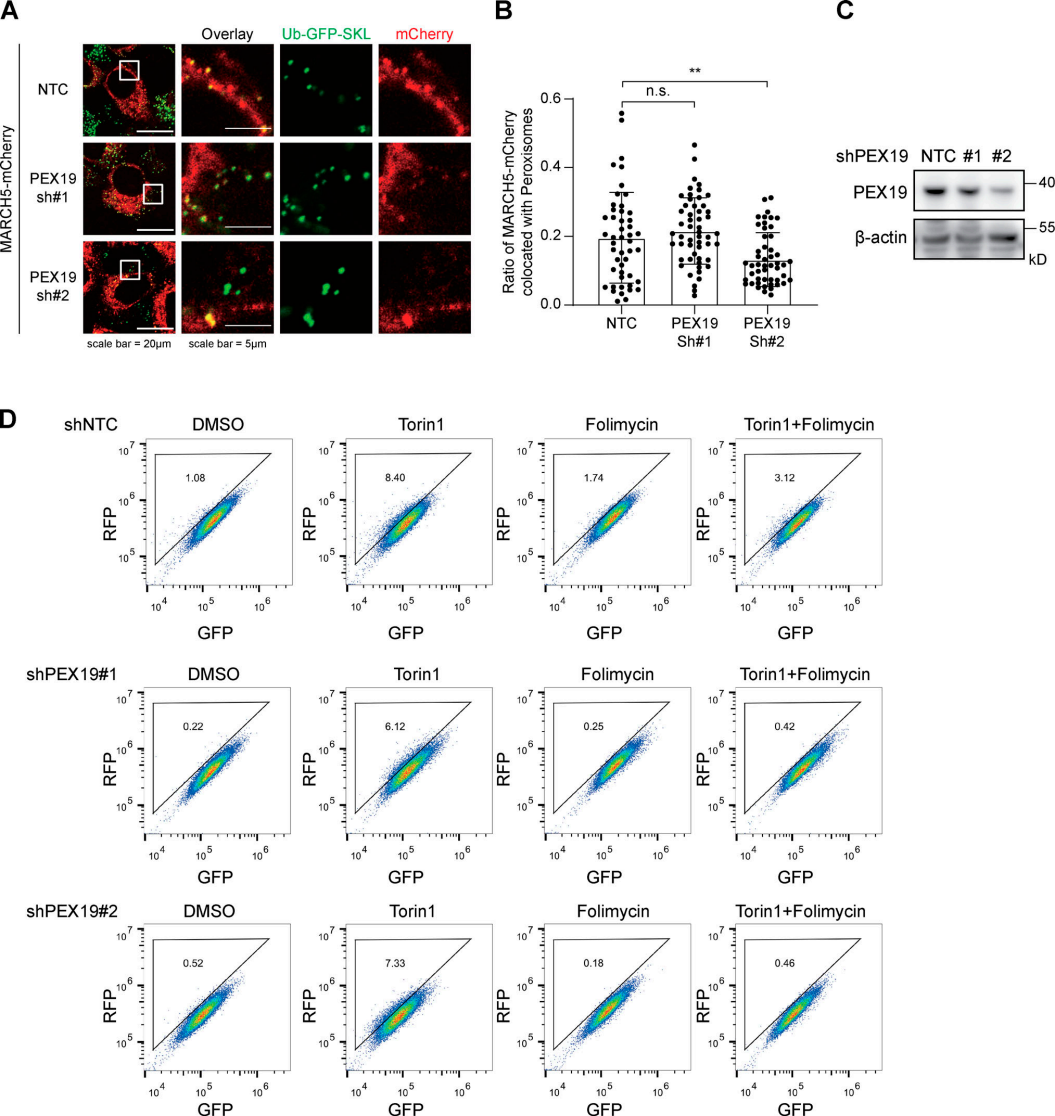
**The effect of knocking down PEX19. (A)** Representative images of WT HeLa cells expressing MARCH5-mCherry and Ub-GFP-SKL. Endogenous PEX19 was knocked down by using shRNA. **(B)** Percentage of MARCH5-mCherry colocalized with peroxisome from A was calculated in ImageJ using Mander’s colocalization coefficients. More than 50 cells were counted. Error bars indicate SD. **, P < 0.01; n.s., not significant (one-way ANOVA and Tukey’s multiple comparisons test). **(C)** The knockdown effect of PEX19 shRNAs was verified at the protein level. **(D)** RFP-GFP-SKL HeLa cells transfected with two different shRNAs targeting PEX19 were treated with Torin1 (1 μM for 24 h), folimycin (10 nM for 24 h), or a combination of the two. Cells were then analyzed by flow cytometry.

To further validate the interaction between MARCH5 and PEX19, we examined the localization of these two proteins. In the absence of overexpressed PEX19, MARCH5-Myc shows a compartmentalized localization, similar to the C-terminal Myc-tagged mitochondrial protein TOMM20 (TOMM20-Myc; Fig. 2 C). When V5-PEX19 is overexpressed, MARCH5 is sequestered in the cytosol, showing a similar distribution pattern as PEX19. We fused a nuclear localization signal (NLS; amino acid sequence MAPKKKRKVGDGS) peptide to the N-terminus of PEX19. NLS-V5-PEX19 mainly located in the nucleus, which also recruited MARCH5-Myc into the nucleus (Fig. 2 C). As a control, TOMM20-Myc localization was not affected by the PEX19 location. We also checked the impact of PEX19 knockdown on the location of MARCH5 in peroxisomes. PEX19 was knocked down with shRNAs. With the reduced PEX19 protein (Fig. S2 C), the percentage of MARCH5-mCherry colocalizing with peroxisomes was also reduced (Fig. S2, A and B), suggesting a critical role of PEX19 for MARCH5 peroxisomal location.

We also examined the interaction between MARCH5 and PEX3. PEX3 bound to the full-length MARCH5 (Fig. 2 D), and the association depended on the TM region on MARCH5 (Fig. 2 E). PEX3 is also a membrane-bound protein with an N-terminal TM helix. To examine the possibility that PEX3 and MARCH5 associate via the TM region, we generated a cytosolic PEX3 (residues 46–373). PEX3(46–373) maintained PEX19 binding capacity but could not localize to the peroxisome. PEX3(46–373) was also coimmunoprecipitated with MARCH5-Myc (Fig. 2 F), suggesting that the interaction does not depend on the N-terminal TM helix on PEX3. We also examined the colocalization of MARCH5 and PEX3. When MARCH5-mCherry and PEX3-YFP were overexpressed in cells, mCherry and YFP signals had partial overlaps (Fig. 2 G). Compared with the overwhelming colocalization of overexpressed MARCH5 and PEX19, the interactions between MARCH5 and PEX3 were limited on the peroxisome surface (Fig. 2 G, white arrows).

### MARCH5 partially locates on peroxisomes

**Figure 3.**
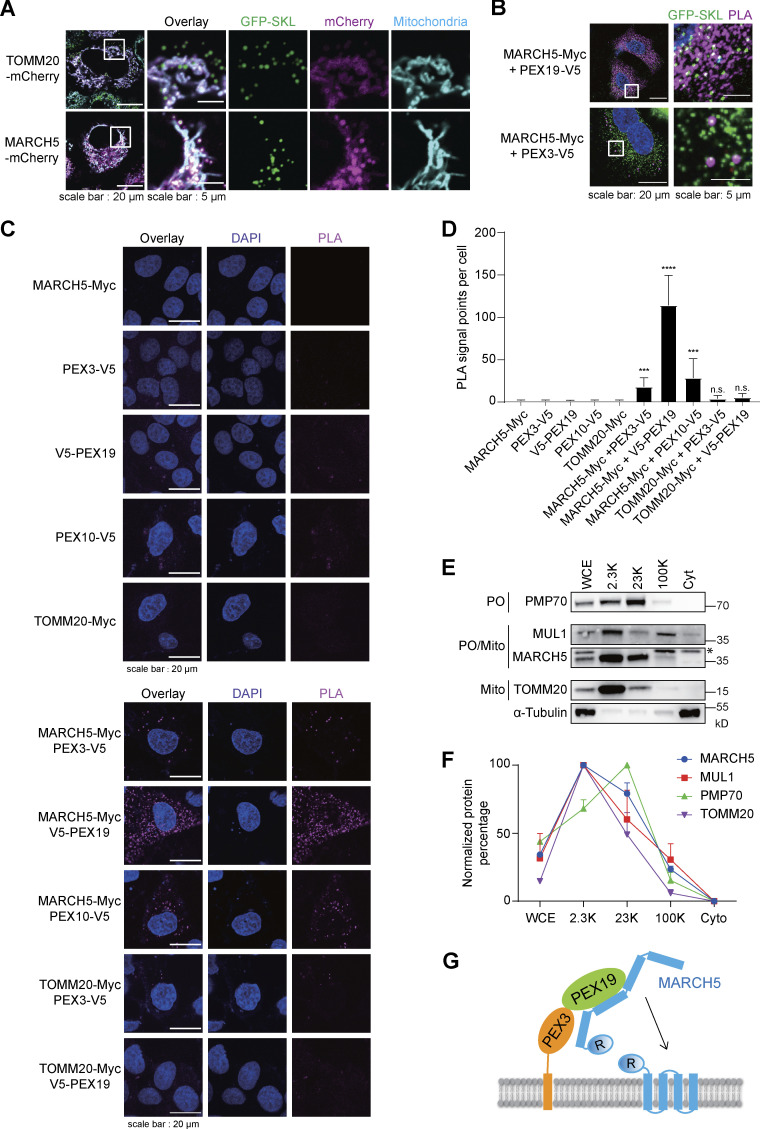
**MARCH5 partially locates on peroxisomes. (A)** HeLa cells stably expressing GFP-SKL were transfected with MARCH5-mCherry or TOMM20-mCherry for 36 h. Mitochondria were stained with MitoTracker Deep Red (50 nM) for 30 min. Cells were analyzed with confocal microscopy. **(B)** PLA was performed with primary antibodies against Myc and V5. HeLa cells stably expressing GFP-SKL were transfected with PEX3-V5, PEX19-V5, and MARCH5-Myc as indicated for 24 h. **(C)** WT HeLa cells were transfected with the indicated constructs for 24 h, and PLA was performed with primary antibodies against Myc and V5. **(D)** PLA signal points in C were calculated and analyzed in ImageJ. Approximately 30 cells were counted in each condition. Error bar represents SD. ***, P < 0.001; ****, P < 0.0001; n.s., not significant (one-way ANOVA and Tukey’s multiple comparisons test). **(E)** Fractionation assay for 293T cells. 5 μg of each fraction isolated from cells was analyzed with IB using the indicated antibodies. The asterisk indicates the nonspecific band. 2.3K, 23K, and 100K, precipitates after centrifugation at indicated speeds; Cyt, supernatants after centrifugation at 100,000 *g*; Mito, mitochondrion; PO, peroxisome; WCE, whole-cell extraction. **(F)** Normalized protein level showing relative protein enrichment in different fractions. Band intensity in E was measured with ImageJ and normalized. Values are mean ± SD, *n* = 3 independent experiments. **(G)** A model for peroxisomal localization of MARCH5 mediated by PEX19 and PEX3. PEX19 binds to the TM region on newly synthesized MARCH5 in the cytosol and transfers it onto the peroxisome surface via PEX3. R, RING domain.

We further examined MARCH5 cellular location by costaining MARCH5 with peroxisomes and mitochondria. When MARCH5 was overexpressed with the mCherry tag, most colocalized with mitochondria, and some were observed on peroxisomes (Fig. 3 A). As a control, overexpressed TOMM20-mCherry colocalized with mitochondria only. To investigate the interaction between MARCH5 and PEX19 in situ, we used the proximity ligation assay (PLA). In PLA, two interacting proteins are recognized by primary antibodies, which are then recognized by detection antibodies conjugated to specific oligonucleotides for polymerase-based amplifications to generate fluorescent signals. MARCH5/PEX19 interactions occurred in the cytosol with strong PLA signals, while MARCH5/PEX3 interactions occurred sporadically in cells but exclusively colocalized with peroxisomes (Fig. 3 B). Biochemically, we show that MARCH5-Myc interacts with PEX3 and PEX19 (Fig. 2), but that does not exclude the possibility that mitochondrial surface protein MARCH5 interacts with the peroxisomal surface protein. More PLA was used to further support that MARCH5-Myc is on the peroxisomal surface. First, other overexpressed mitochondrial proteins, like TOMM20, did not interact with PEX19 or PEX3 (Fig. 3, C and D). Second, MARCH5 was in proximity with another peroxisomal protein, PEX10, further confirming its peroxisome localization (Fig. 3, C and D).

We sought whether endogenous MARCH5 also locates on peroxisomes. Due to the lack of a suitable antibody for MARCH5 immunofluorescence staining, we used a fractionation experiment to roughly separate peroxisomes from other organelles by centrifugation. The peroxisome marker protein PMP70 was mainly enriched in the 23K fraction, while mitochondrion marker TOMM20 was mainly enriched in the 2.3K fraction. The mitochondria/peroxisome dual-localization protein MUL1 was also examined (Fig. 3 E). In the 23K fraction, the percentage of endogenous MARCH5 and MUL1 was higher than the typical mitochondrial protein TOMM20 and lower than peroxisomal protein PMP70 (Fig. 3 F), suggesting MARCH5 as another mitochondria/peroxisome dual-localization protein. Considering that MARCH5 interacted with PEX19 and PEX3 (Fig. 2), these data fit a model of PEX19/PEX3-mediated MARCH5 peroxisome localization (Fig. 3 G).

### MARCH5 binds and ubiquitinates PMP70 and PEX3

**Figure 4.**
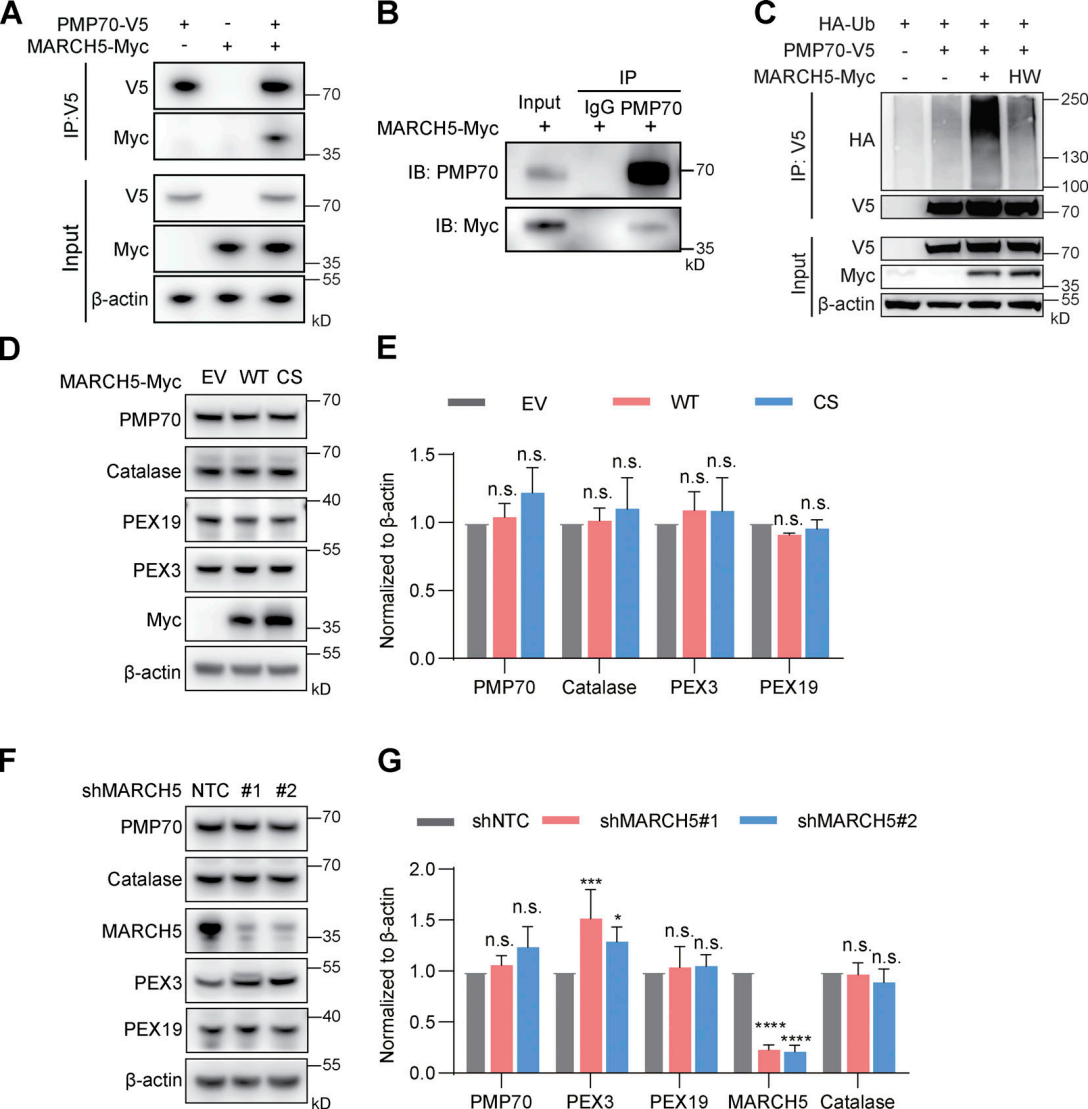
**MARCH5 binds and ubiquitinates PMP70. (A)** IP was performed with anti-V5 antibody in HEK293T cells transfected with PMP70-V5 and MARCH5-Myc, followed by IB for V5, Myc, and β-actin. **(B)** HeLa cells were transfected with MARCH5-Myc plasmids for 48 h. After 10 μM MG132 treatment for 6 h, IP was performed with anti-PMP70 antibody, followed by IB for PMP70 and Myc. **(C)** HEK293T cells were transfected with HA-Ub, PMP70-V5, MARCH5-Myc, and MARCH5(HW)-Myc plasmids for 48 h. After 10 μM MG132 treatment for 6 h, cell lysates were immunoprecipitated with anti-V5 antibody and then immunoblotted with indicated antibodies. **(D)** HeLa cells were transfected with empty vector (EV), MARCH5-Myc, or catalytically inactive MARCH5-CS-Myc plasmids for 48 h. The cell lysates were immunoblotted with indicated antibodies. **(E)** The protein levels in D were quantified with ImageJ, then normalized to β-actin. Values are mean ± SD, *n* = 3 independent experiments. n.s., not significant (P > 0.05) by two-tailed Student’s *t* test. **(F)** HeLa cells were transfected with two different shRNAs targeting MARCH5. The cell lysates were immunoblotted with indicated antibodies. **(G)** The protein levels in F were quantified with ImageJ, then normalized to β-actin. Values are mean ± SD, *n* = 3 independent experiments. *, P < 0.05; ***, P < 0.001; ****, P < 0.0001; n.s., not significant (P > 0.05) by two-tailed Student’s *t* test.

Next, we addressed the function of MARCH5 on peroxisomes. In addition to PEX19 and PEX3, we identified PMP70 (also referred to as ABCD3) by MS as one potential MARCH5-interacting protein (Fig. 1, D and E). We validated the interaction between MARCH5 and PMP70 by coimmunoprecipitation experiments. When V5-tagged PMP70 and Myc-tagged MARCH5 were coexpressed in cells, immunoprecipitation (IP) of PMP70-V5 captured associated MARCH5-Myc (Fig. 4 A). In cells expressing MARCH5-Myc, MARCH5-Myc was coimmunoprecipitated with endogenous PMP70 (Fig. 4 B). We further set up a ubiquitination assay with HA-tagged ubiquitin (HA-Ub) and V5 tagged PMP70. The ubiquitination level of PMP70-V5 was significantly enhanced in the presence of overexpressed MARCH5-Myc but not with the enzymatically inactive MARCH5(HW)-Myc (Fig. 4 C).

Ubiquitination usually leads to protein degradation. However, in the presence of overexpressed MARCH5-Myc, the protein level of PMP70-V5 did not show any significant change (Fig. 4 C). We further examined the endogenous PMP70 level with overexpressed MARCH5-Myc (Fig. 4, D and E) or reduced MARCH5 (Fig. 4, F and G). The levels of PMP70 and other peroxisomal proteins were mostly unaffected, suggesting that MARCH5-mediated ubiquitination of PMP70 might be at a low level in normal conditions.

**Figure S3.**
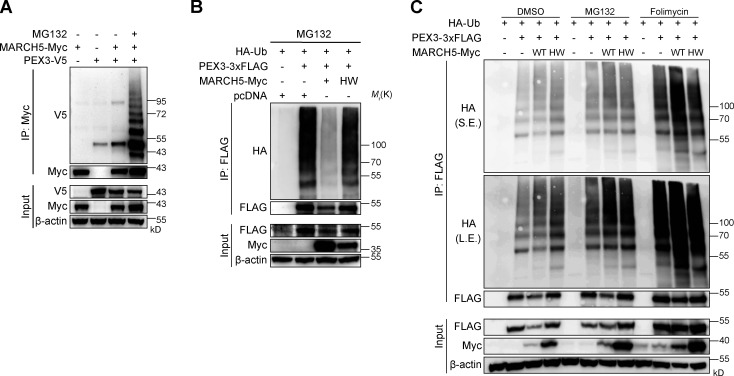
**MARCH5 ubiquitinates PEX3. (A)** HeLa cells were cotransfected with MARCH5-Myc and PEX3-V5 plasmids for 18 h and treated with 10 μM MG132 for 6 h in the indicated sample. IP and IBs were performed with anti-Myc and anti-V5 antibodies. **(B)** MARCH5 KO HeLa cells were cotransfected with HA-Ub, PEX3-3×FLAG, and MARCH5-Myc for 18 h and treated with MG132 (10 μM) for 6 h. IP was performed with anti-FLAG antibody and followed by IB with indicated antibodies. **(C)** 293FT cells were cotransfected with HA-Ub, PEX3-3×FLAG, and MARCH5-Myc for 48 h. Cells were treated with DMSO, MG132 (10 μM for 6 h), or folimycin (10 nM for 24 h). IP was performed with anti-FLAG antibody and followed by IB with indicated antibodies. L.E., long exposure; S.E., short exposure.

Among those peroxisomal proteins, PEX3 level significantly increased when MARCH5 was knocked down (Fig. 4 G). The addition of proteasome inhibitor MG132 enhanced the level of MARCH5-associated PEX3 (Fig. S3 A) but could not block the degradation of PEX3 in the presence of WT MARCH5 (Fig. S3 B). We performed a ubiquitination assay to see whether MARCH5 is responsible for PEX3 ubiquitination. WT cells were cotransfected with HA-Ub, PEX3-3×FLAG, and MARCH5-Myc and then treated with either proteasome inhibitor MG132 or lysosome inhibitor folimycin. The ubiquitination of PEX3-3×FLAG increased in the presence of WT MARCH5 but not with MARCH5(HW). The addition of MG132 slightly increased the ubiquitin signal, while the addition of folimycin significantly enhanced the ubiquitin signal (Fig. S3 C). This result suggests that MARCH5 ubiquitinates PEX3 when both proteins are overexpressed. The ubiquitinated PEX3 is mainly degraded by lysosome and partly degraded in proteasome in this condition.

### Torin1 induces PMP70 ubiquitination and pexophagy

It was previously reported that ubiquitin signals autophagic degradation of peroxisomes, and the ubiquitination of PMP70 mediates pexophagy during starvation (Kim et al., 2008; Sargent et al., 2016). We wondered whether MARCH5 plays a role in this process. To investigate the role of MARCH5-mediated PMP70 ubiquitination, we first established a condition of PMP70 ubiquitination in pexophagy.

**Figure 5.**
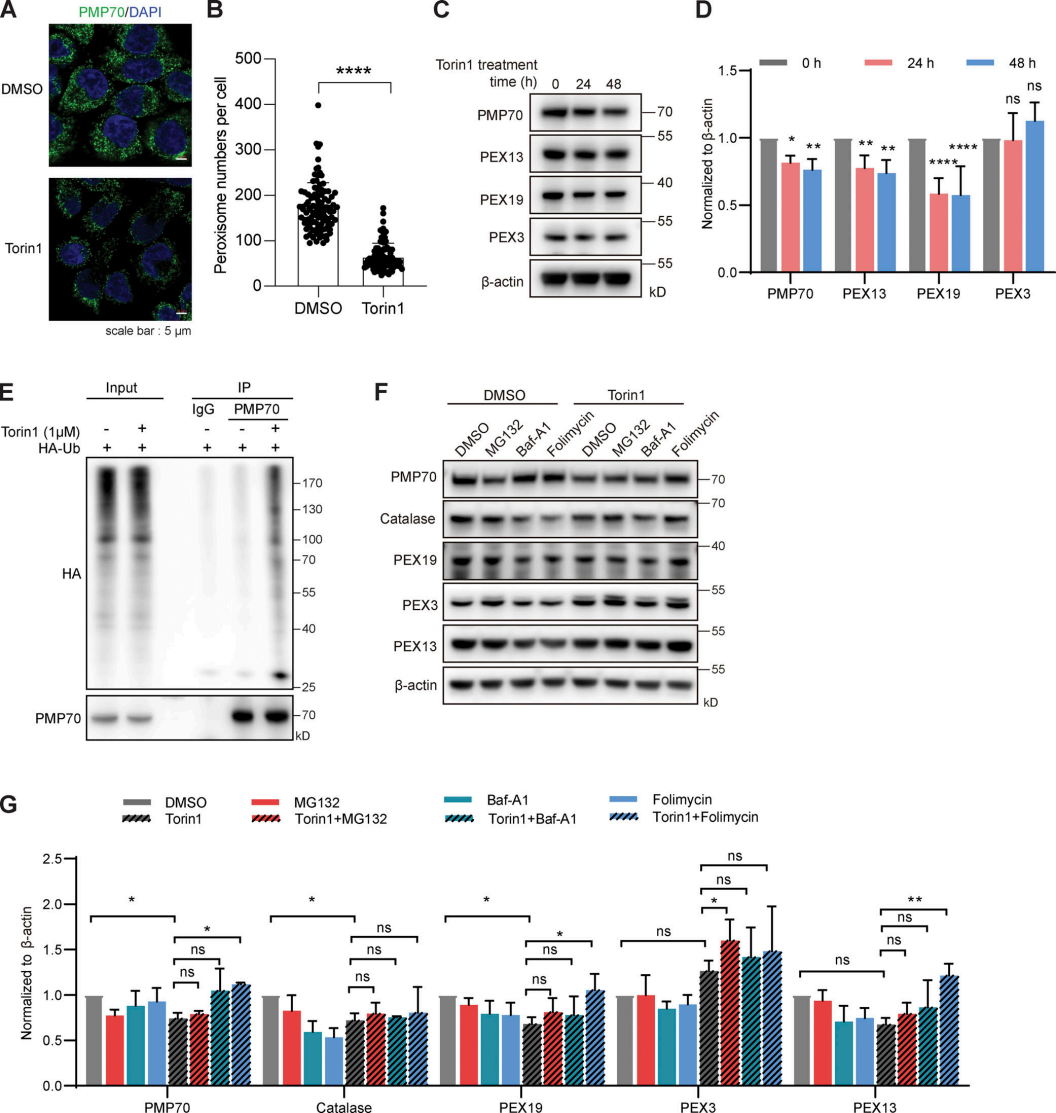
**Torin1 induces PMP70 ubiquitination and degradation. (A)** Representative fluorescence images of HeLa cells treated with Torin1 (1 μM) or DMSO for 24 h as indicated and immunostained for PMP70. **(B)** Quantification of peroxisome numbers (green puncta) in cells treated as in A. Values are mean ± SD, *n* = 3 independent experiments, calculated using >90 cells. **(C)** HeLa cells were treated with Torin1 for the indicated time. The cell lysates were immunoblotted with the indicated antibodies. **(D)** Quantification of the protein levels in C. **(E)** HeLa cells were transfected with HA-Ub for 24 h and then treated with Torin1 (1 μM) for 24 h and MG132 (10 μM) for 4 h. Cell lysates were then subjected to a ubiquitination assay and immunoprecipitated with anti-PMP70 antibody, followed by WB analysis. **(F)** HeLa cells were treated with DMSO, Torin1 (1 μM for 24 h), MG132 (10 μM for 4 h), Baf-A1 (40 nM for 24 h), and folimycin (10 nM for 24 h) as indicated and immunoblotted with PMP70, catalase, PEX19, PEX3, PEX13, and β-actin antibodies. **(G)** Quantification of F. In D and G, the protein levels were quantified with ImageJ, then normalized to β-actin. Values are mean ± SD, *n* = 3 independent experiments. *, P < 0.05; **, P < 0.01; ****, P < 0.0001; ns, P > 0.05 by two-tailed Student’s *t* test.

**Figure S4.**
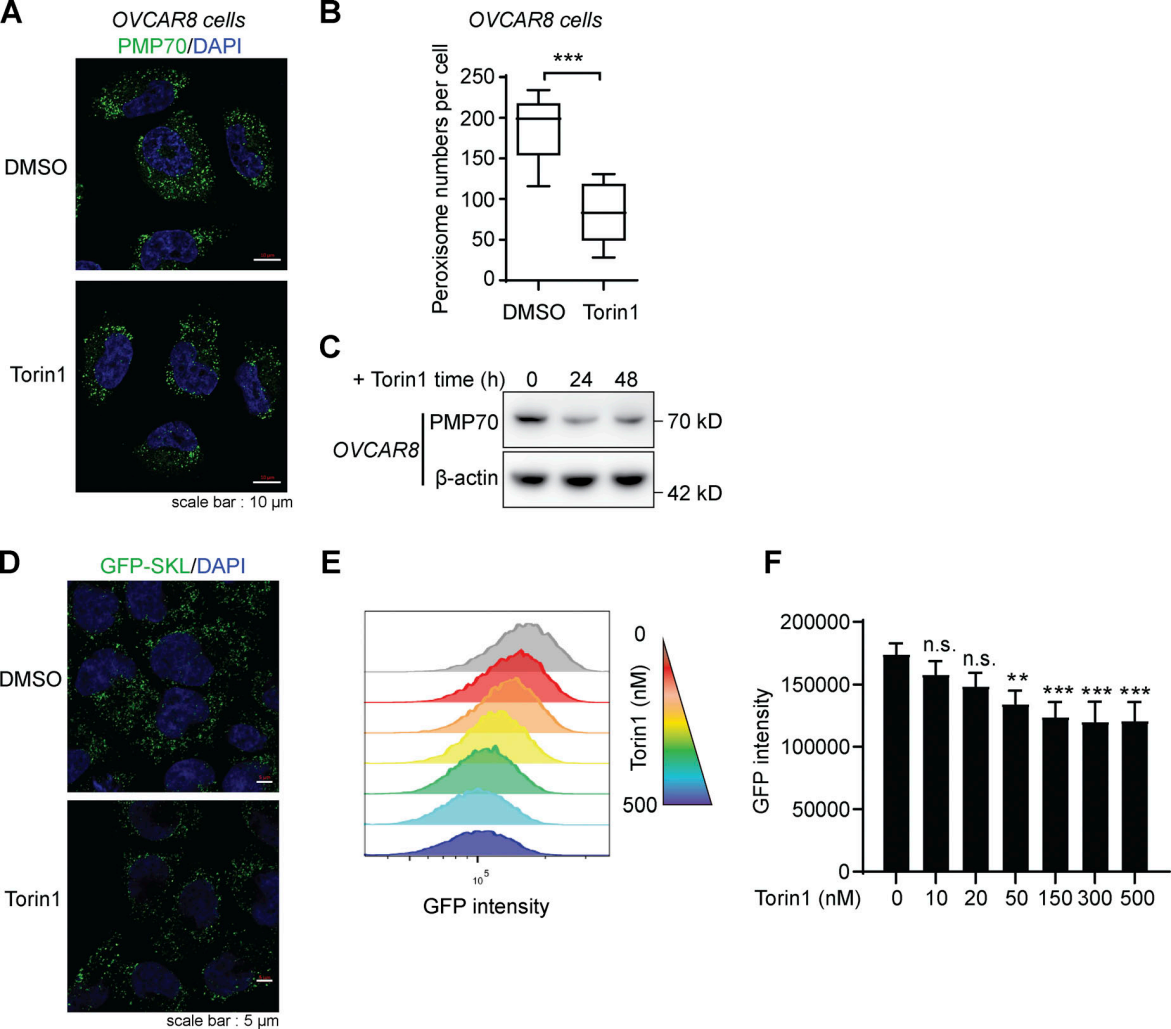
**Torin1 induces pexophagy in different cells. (A)** Representative fluorescence images of *OVCAR8* cells treated with Torin1 (1 μM) or DMSO for 24 h as indicated and immunostained for PMP70. Scale bars: 10 µm. **(B)** Quantification of peroxisome numbers (green puncta) in cells treated in A. Values are mean ± SD, *n* = 3 independent experiments, calculated using >90 cells. ***, P < 0.001 by two-tailed Student’s *t* test. **(C)** *OVCAR8* cells were treated with DMSO or Torin1 for the indicated time. The cell lysates were analyzed by WB and immunoblotted with PMP70 and β-actin antibodies. **(D)** HeLa cells stably expressing GFP-SKL were treated with DMSO or Torin1 (1 μM) for 24 h. Cells were then fixed and examined by microscopy to detect GFP signal. Scale bars: 5 µm. **(E)** HeLa cells stably expressing GFP-SKL were treated with or without Torin1 (1 μM for 24 h). Cells were then analyzed by flow cytometry. **(F)** Quantification of GFP intensity in E. Values are mean ± SD, *n* = 3 independent experiments. **, P < 0.01; ***, P < 0.001; n.s., not significant by one-way ANOVA and Dunnett’s multiple comparisons test.

To induce pexophagy, we treated HeLa cells with the mTOR inhibitor Torin1. With the treatment of Torin1, the cells showed a reduced number of peroxisomes by PMP70 staining (Fig. 5, A and B). Similar results could be observed in another cell line, OVCAR8 (Fig. S4, A–C). Consistently, the PMP70 level was reduced (Fig. 5, C and D), while its ubiquitination level increased with Torin1 treatment (Fig. 5 E). Other peroxisome-related proteins were also examined. PEX13 and PEX19 showed reduced levels upon Torin1 treatment, while PEX3 remained unchanged (Fig. 5, C and D). We used an alternative peroxisome marker, GFP-Ser-Lys-Leu (SKL), to indicate peroxisomes. The C-terminus of GFP was fused to the peroxisomal matrix–targeting signal peptide SKL to direct GFP to peroxisomes. Torin1 treatment also significantly reduced the GFP signal compared with the control (Fig. S4, D–F). PMP70, PEX13, and the matrix protein GFP-SKL all decreased upon Torin1 treatment, suggesting the degradation of the organelle.

Individual proteins are usually degraded via proteasomes, while organelles are degraded via lysosomes. We added proteasome inhibitor MG132 or lysosome inhibitors (Baf-A1 or folimycin) with Torin1 treatment. PMP70 degradation was not inhibited by MG132 but could be blocked by folimycin (Fig. 5, F and G). Similarly, the degradation of PEX13 and PEX19 could be blocked by folimycin but not by MG132. These results suggest that the peroxisomes are targeted for lysosomal degradation.

**Figure 6.**
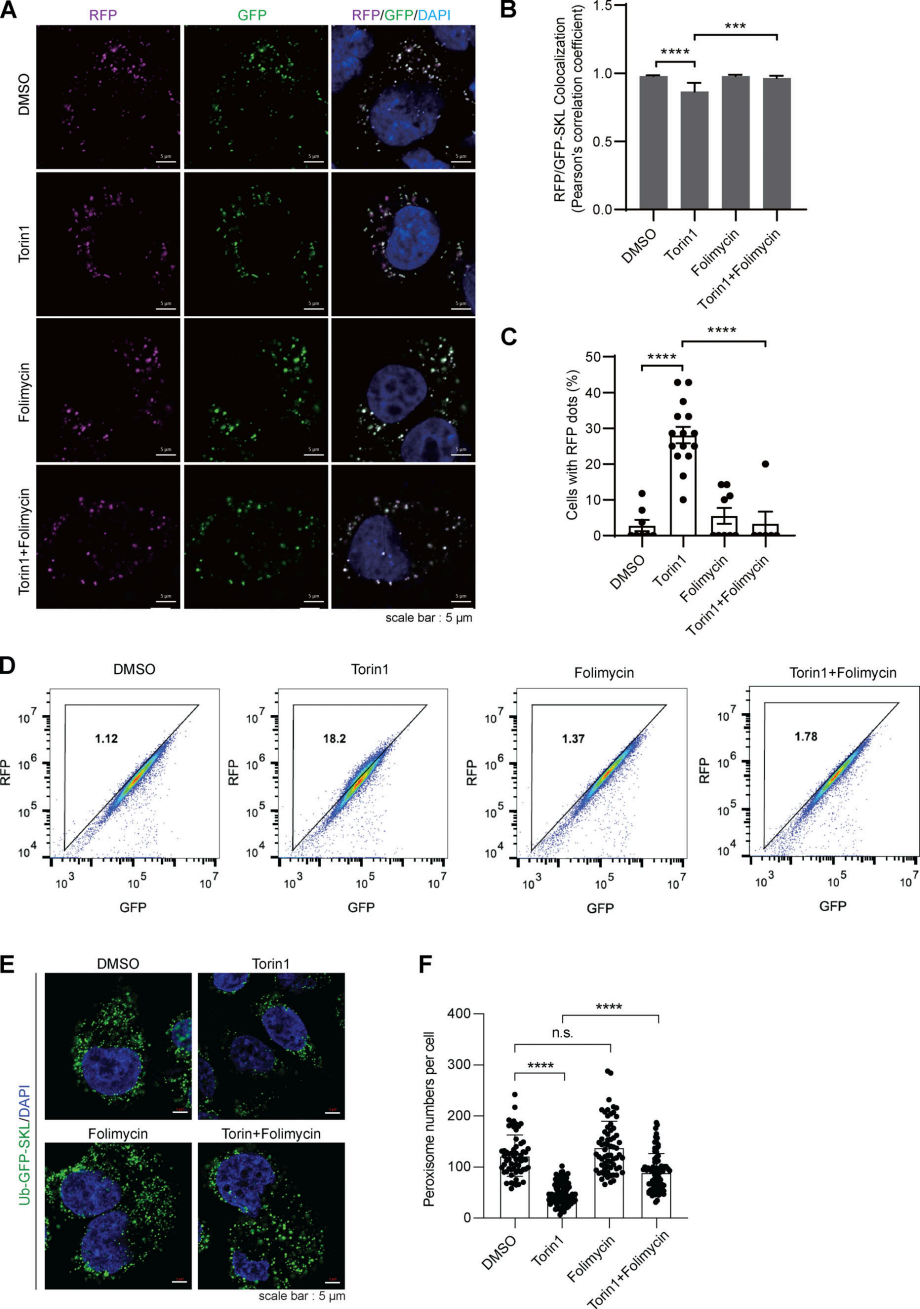
**Torin 1 induces pexophagy. (A)** HeLa cells stably expressing RFP-GFP-SKL were treated with DMSO or Torin1 (1 μM) for 24 h. Cells were then fixed and examined by microscopy to detect RFP and GFP signals. Scale bars: 5 µm. **(B)** Pearson’s correlation coefficients showing the colocalization between RFP-SKL and GFP-SKL derived from A. Values are mean ± SD, calculated using >50 cells. ***, P < 0.001; ****, P < 0.0001 by one-way ANOVA and Tukey’s multiple comparisons test. **(C)** Percentage of cells showing red fluorescence that are not overlapping with GFP. Values are mean ± SD, calculated using >50 cells. ****, P < 0.0001 by one-way ANOVA and Tukey’s multiple comparisons test. **(D)** HeLa cells stably expressing RFP-GFP-SKL were treated with Torin1 (1 μM for 24 h), folimycin (10 nM for 24 h), or a combination of the two. Cells were then analyzed by flow cytometry. **(E)** Hela cells stably expressing Ub-GFP-SKL were treated with Torin1 (1 μM for 24 h), folimycin (10 nM for 24 h), or a combination of the two. Cells were then fixed and examined by microscopy to detect GFP signal. Scale bars: 5 µm. **(F)** Quantification of peroxisome numbers by counting GFP-positive spots in E. Values are mean ± SD, *n* = 3 independent experiments, calculated using >60 cells. ****, P < 0.0001; n.s., not significant by one-way ANOVA and Tukey’s multiple comparisons test.

To further confirm the induction of pexophagy, cells expressing peroxisome targeting RFP-GFP-SKL were treated with Torin1 with or without folimycin. In the presence of Torin1, the GFP signal was quenched in acidic lysosomes, leaving fewer correlated RFP/GFP signals (Fig. 6, A and B). More cells contained RFP signals that were not overlapping with GFP (Fig. 6 C). In addition, flow cytometry analysis revealed that more cells had a higher RFP/GFP ratio in the group treated with Torin1. Folimycin, which inhibits the acidification of lysosomes, rescued the RFP/GFP ratio in Torin1-treated cells (Fig. 6 D). Furthermore, in the presence of folimycin, the peroxisome numbers remain unchanged with Torin1 treatment (Fig. 6, E and F). Therefore, mTOR inhibition by Torin1 induces PMP70 ubiquitination and pexophagy.

### MARCH5 is required for Torin1-induced PMP70 ubiquitination and pexophagy

**Figure S5.**
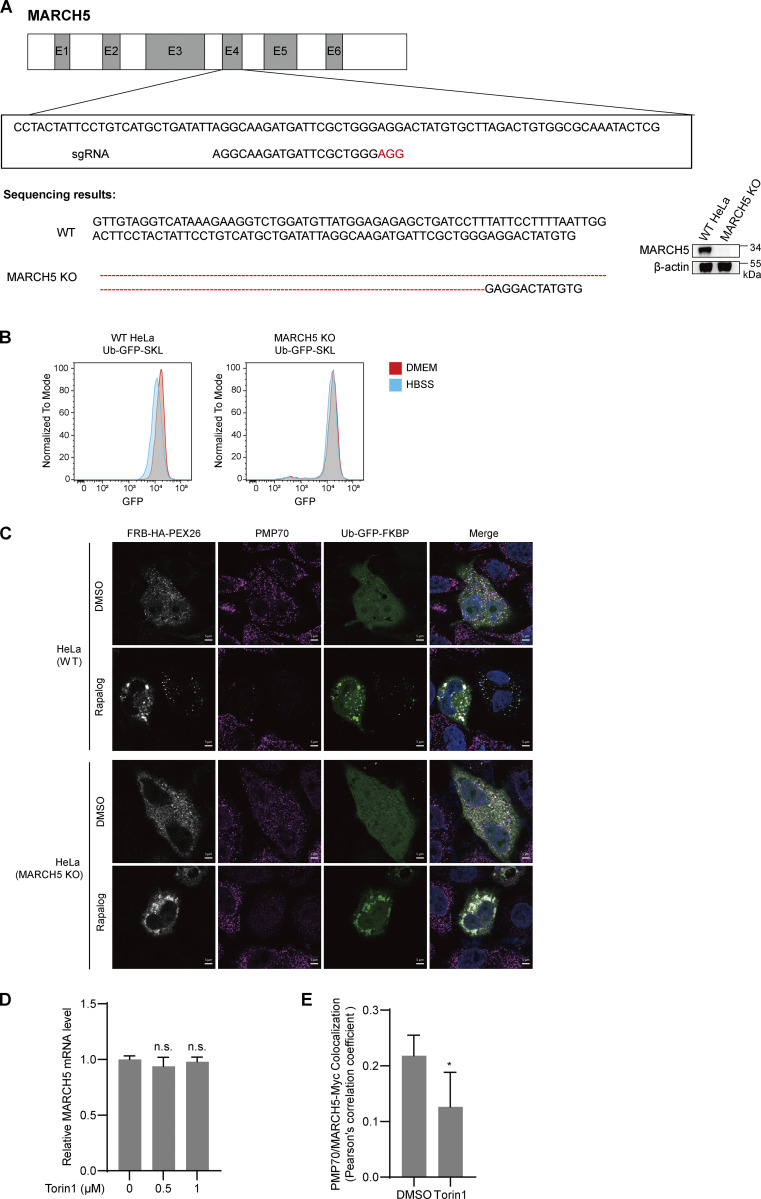
**Other supplementary information. (A)** Schematic of the genome-editing strategy to knock out endogenous MARCH5 in HeLa cells. Exons 1–6 (E1-E6) are indicated. Three designed sgRNAs were tested initially, and the validated sgRNA confirmed by sequencing, as shown. Protospacer adjacent motif sequences are depicted in red. The KO of MARCH5 is confirmed by both Sanger sequencing (left) and IBs (right). **(B)** WT or MARCH5 KO HeLa cells expressing Ub-GFP-SKL were cultured in DMEM (supplemented with 10% FBS) or HBSS for 24 h. GFP signal was analyzed with FACS. **(C)** Representative images for Fig 9, D and E. WT or MARCH5 KO HeLa cells were transfected with Ub-GFP-FKBP and FRB-HA-PEX26 for 24 h. Cells were treated with DMSO or rapalog for 24 h, fixed, immunostained for PMP70, and imaged with confocal microscopy. Scale bars: 5 µm. **(D)** HeLa cells were treated with Torin1 (1 μM) for the indicated time. MARCH5 mRNA levels were detected with quantitative PCR assay. Values are mean ± SD, *n* = 3 independent experiments. n.s., not significant (P > 0.05) by two-tailed Student’s *t* test. **(E)** HeLa cells were transfected with MARCH5-Myc plasmid for 24 h, then with or without Torin1 (1 μM) were added for an additional 24 h before harvesting. Cells were then fixed and immunostained for PMP70 (Alexa Fluor 488, green) and Myc (Alexa Fluor 555, red) and examined by microscopy to detect GFP and RFP signals. Pearson’s correlation coefficients show the colocalization between PMP70 and MARCH5-Myc derived from above. Values are mean ± SD *, P < 0.05 by two-tailed Student’s *t* test.

**Figure 7.**
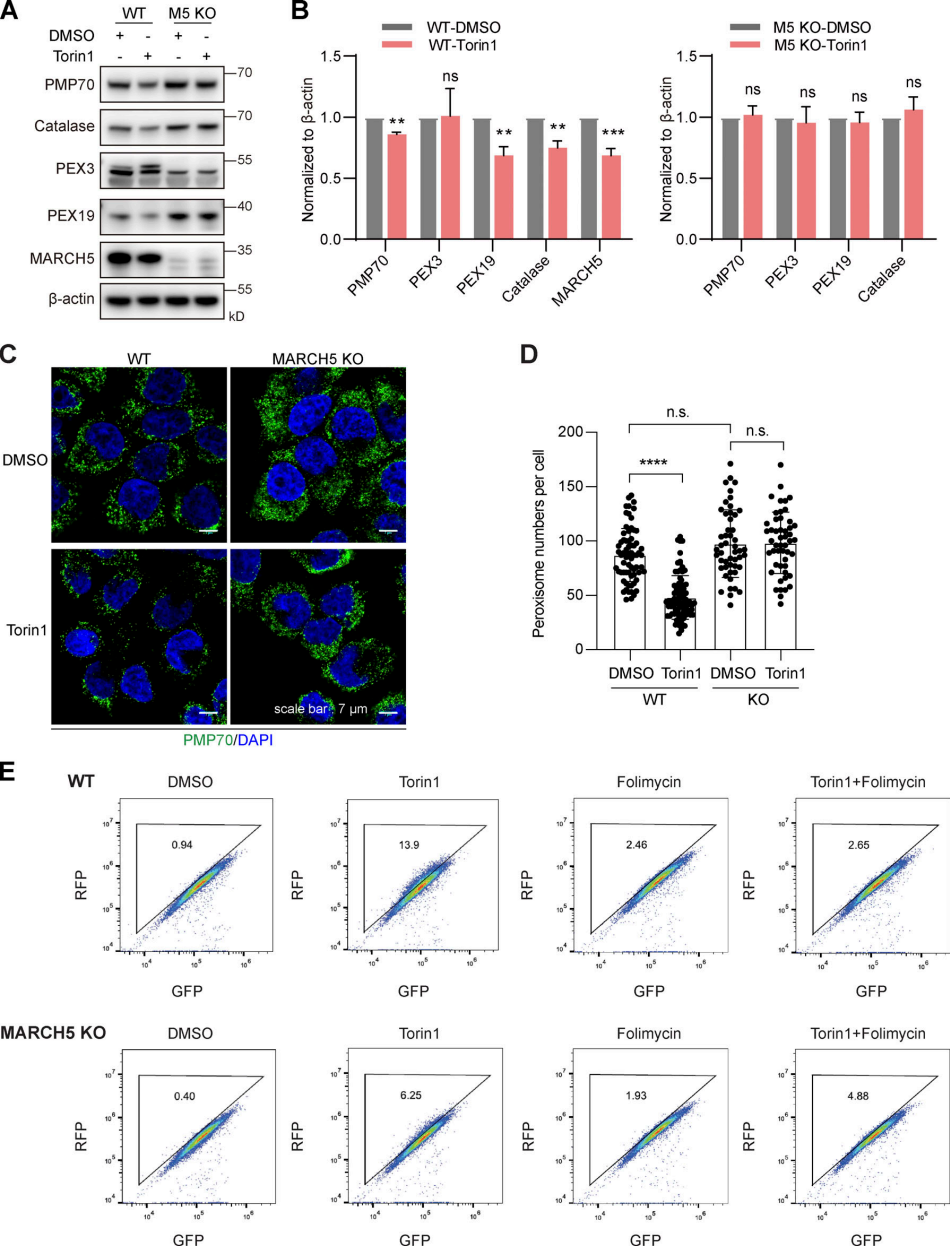
**KO of MARCH5 blocks Torin1-induced PMP70 degradation and pexophagy. (A)** WT and MARCH5 KO HeLa cells were treated with Torin1 (1 μM) or DMSO for 24 h and immunoblotted with indicated antibodies. **(B)** The protein levels in A were quantified with ImageJ, then normalized to β-actin. Values are mean ± SD, *n* = 3 independent experiments. **, P < 0.01; ***, P < 0.001; ns, P > 0.05 by two-tailed Student’s *t* test. **(C)** WT and MARCH5 KO HeLa cells were treated with Torin1 (1 μM) or DMSO for 24 h as indicated and immunostained for PMP70. Scale bars: 7 µm. **(D)** Quantification of peroxisome numbers (green puncta) in cells treated in C. Values are mean ± SD, *n* = 3 independent experiments, calculated using >50 cells. ****, P < 0.0001; n.s., not significant by one-way ANOVA and Tukey’s multiple comparisons test. **(E)** WT and MARCH5 KO HeLa cells stably expressing RFP-GFP-SKL were treated with Torin1 (1 μM for 24 h), folimycin (10 nM for 24 h), or a combination of the two. Cells were then analyzed by flow cytometry.

We next asked whether MARCH5 is involved in Torin1-induced PMP70 ubiquitination and pexophagy. We first generated a MARCH5 knockout (KO) stable HeLa cell line with the CRISPR/Cas9 system (Fig. S5 A). Both WT and MARCH5 KO HeLa cells were treated with 1 μM Torin1 for 24 h. Protein levels of PMP70, catalase, and PEX19 all reduced in WT HeLa cells. However, in MARCH5 KO cells, there was no apparent change of these peroxisome proteins (Fig. 7, A and B). We further assessed the peroxisome numbers in MARCH5 KO cells. Immunofluorescence staining of PMP70 showed that the number of peroxisomes remained unchanged in MARCH5 KO cells with Torin1 treatment (Fig. 7, C and D). Using GFP-RFP-SKL as a pexophagy sensor, flow cytometry analysis showed that the pexophagy was inhibited in MARCH5 KO cells with Torin1 treatment (Fig. 7 E). These results suggest that MARCH5 is a regulator for Torin1-induced pexophagy. We also examined the role of MARCH5 in pexophagy in a starvation condition. Ubiquitin-fused GFP-SKL was used to indicate peroxisome numbers in cells. WT HeLa cells growing in HBSS medium showed decreased GFP signal compared with cells growing in complete medium. However, the GFP signals in MARCH5 KO cells did not respond to the different media (Fig. S5 B).

**Figure 8.**
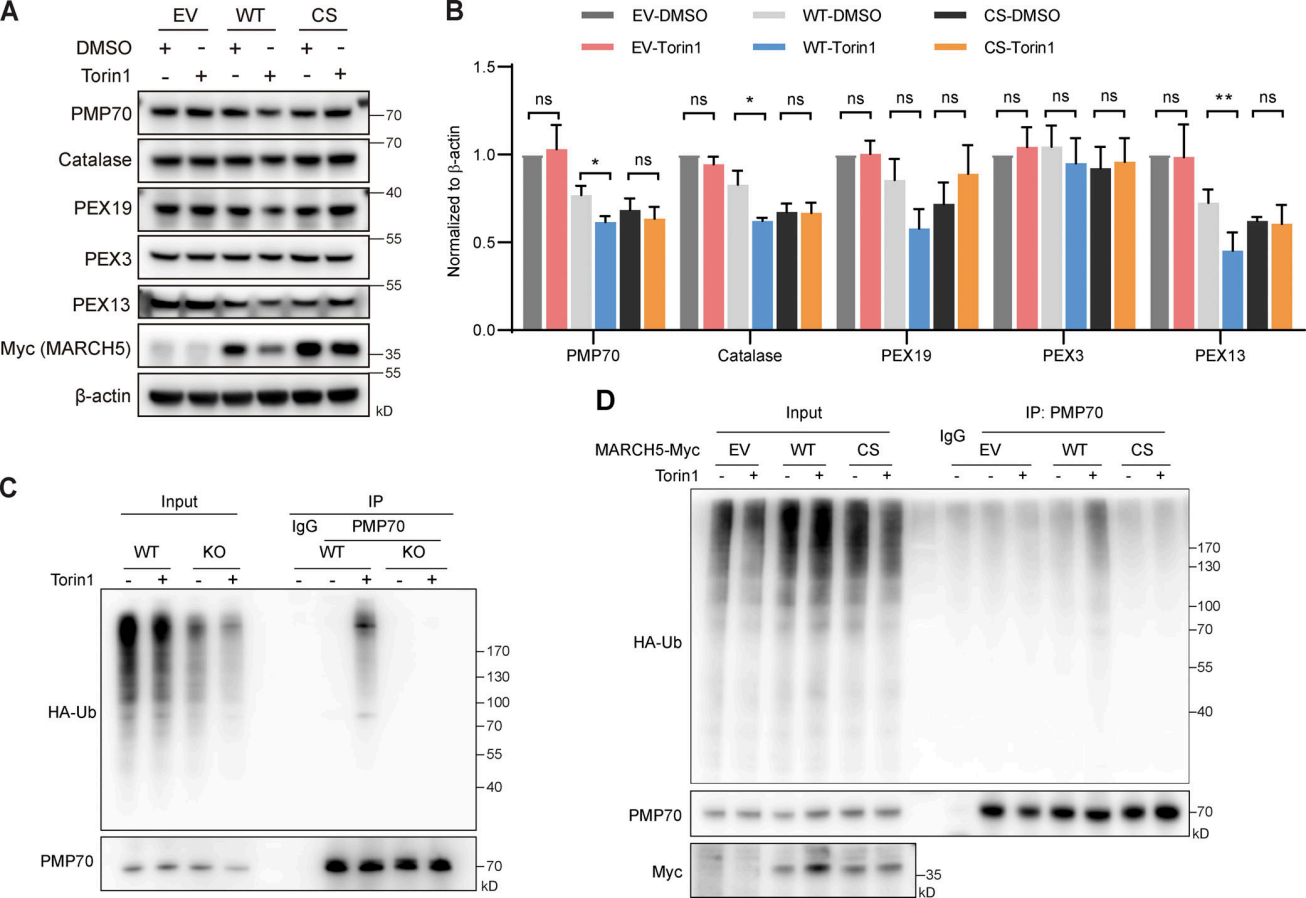
**MARCH5 ubiquitin ligase activity is required for PMP70 ubiquitination and degradation with Torin1 treatment. (A)** MARCH5 KO cells were transfected with empty vector (EV), MARCH5-Myc, or catalytically inactive MARCH5-CS-Myc plasmid for 24 h and treated with Torin1 (1 μM for 24 h), as indicated. The cell lysates were immunoblotted with indicated antibodies. **(B)** The protein levels in A were quantified with ImageJ, then normalized to β-actin. Values are mean ± SD, *n* = 3 independent experiments. *, P < 0.05; **, P < 0.01; ns, P > 0.05 by two-tailed Student’s *t* test. **(C)** WT and MARCH5 KO HeLa cells were transfected with HA-Ub for 24 h and then treated with Torin1 (1 μM for 24 h) and MG132 (10 μM for 4 h). Cell lysates were then subjected to a ubiquitination assay and immunoprecipitated with anti-PMP70 antibody, followed by WB analysis. **(D)** MARCH5 KO cells were transfected with MARCH5-Myc or catalytically inactive MARCH5-CS-Myc and HA-Ub plasmid for 24 h and treated with Torin1 and MG132, as indicated. The ubiquitination assay was performed, and cell lysates were immunoprecipitated with anti-PMP70 antibody, followed by WB analysis.

Ubiquitin ligase activity of MARCH5 is required for the ubiquitination and degradation of PMP70. WT MARCH5-Myc or catalytically inactive MARCH5(C65/68S)-Myc was introduced into MARCH5 KO cells. In the presence of WT MARCH5-Myc, protein levels of PMP70, catalase, and PEX13 reduced with Torin1 treatment (Fig. 8, A and B). MARCH5(C65/68S)-Myc could not induce the degradation of these peroxisomal proteins. We also examined the ubiquitination level of PMP70 in MARCH5 KO cells. Both WT and MARCH5 KO HeLa cells were transfected with HA-Ub and then treated with Torin1. Endogenous PMP70 was enriched by IP, and the ubiquitination status was inspected with anti-HA antibody. Upon Torin1 treatment, the ubiquitination level of PMP70 increased in WT cells but not in MARCH5 KO cells (Fig. 8 C). Expression of MARCH5-Myc in MARCH5 KO cells reestablished the increased ubiquitination of PMP70 upon Torin1 treatment. On the other hand, the catalytically inactive MARCH5(C65/68S)-Myc could not rescue PMP70 ubiquitination (Fig. 8 D). Therefore, the ubiquitin ligase activity of MARCH5 is required for PMP70 ubiquitination during Torin1-induced pexophagy.

### Peroxisomal targeting MARCH5(RING) reestablishes pexophagy

**Figure 9.**
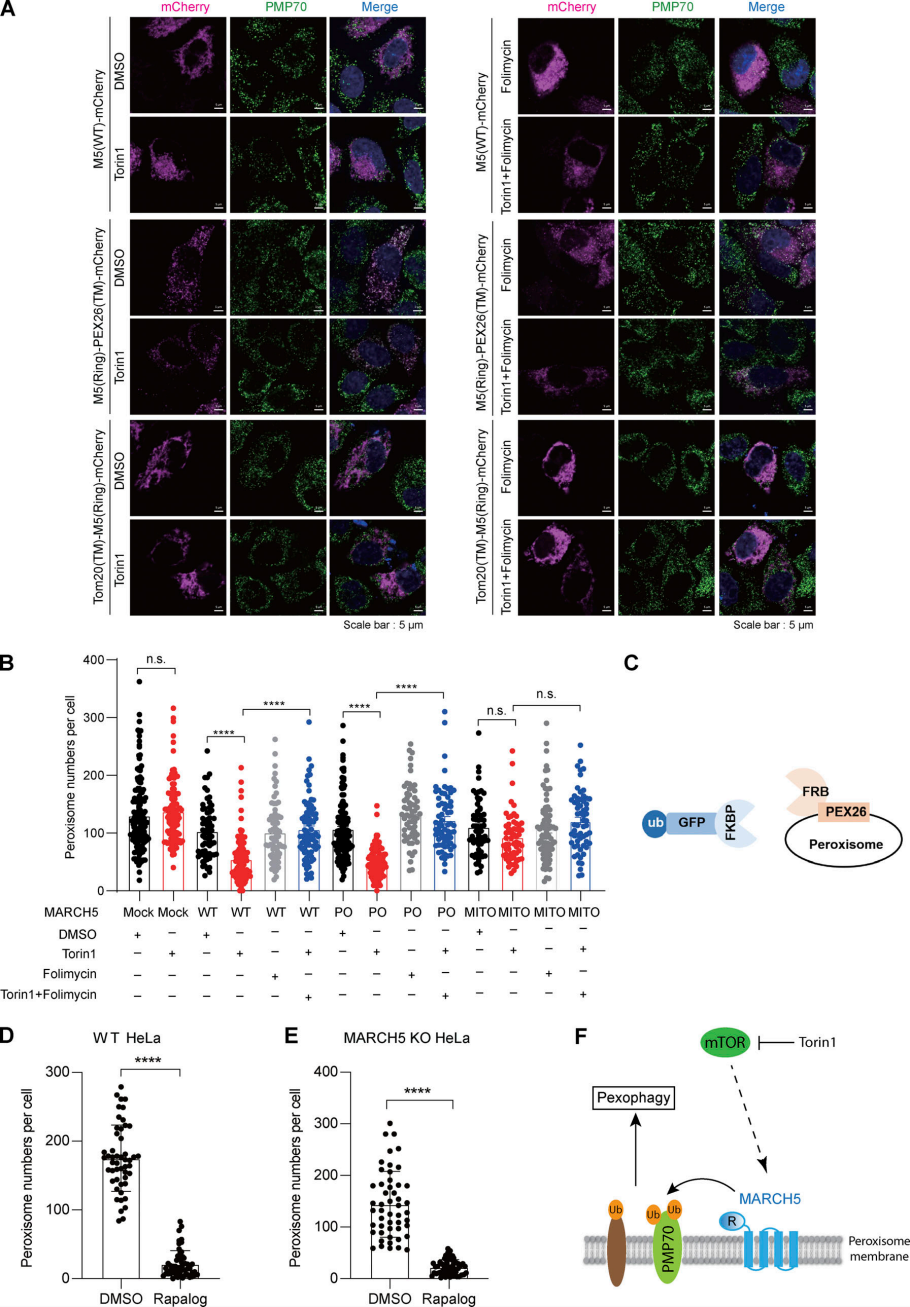
**Peroxisome-localized MARCH5(RING) domain is sufficient to mediate Torin1-induced pexophagy. (A)** MARCH5 KO HeLa cells were transfected with MARCH5(WT)-mCherry, MARCH5(Ring)-mCherry-PEX26(TM) (referred as po-MARCH5) or TOMM20-MARCH5(RING)-mCherry (referred as mito-MARCH5) for 24 h and treated with Torin1 (1 μM for 24 h), folimycin (10 nM for 24 h), or a combination of the two for an additional 24 h. Cells were then fixed and stained for PMP70 (Alexa Fluor 488; green). Scale bars: 5 µm. **(B)** Quantification of peroxisome numbers (green puncta) in cells treated in A. Values are calculated using >50 cells. ****, P < 0.0001; n.s., not significant by one-way ANOVA and Tukey’s multiple comparisons test. **(C)** Schematic of induced peroxisome ubiquitination (ub) using FRB-PEX26 and Ub-GFP-FKBP. **(D and E)** WT HeLa or MARCH5 KO HeLa cells were transfected with Ub-GFP-FKBP and FRB-HA-PEX26 for 24 h. Cells were treated with or without rapalog for 24 h, fixed, immunostained for PMP70, and imaged with confocal microscopy (representative images in Fig. S5). The peroxisome number was quantified. Values are mean ± SD, *n* = 3 independent experiments, calculated using >50 cells. ****, P < 0.0001 by two-tailed Student’s *t* test. **(F)** Model of the role of MARCH5 in Torin1-induced pexophagy. R, RING domain.

MARCH5 is a mitochondrion and peroxisome dual-localized protein, while PMP70 is considered exclusively on peroxisomes. We wondered whether the ubiquitination of PMP70 is performed by peroxisomal MARCH5 or mitochondrial MARCH5. To address this issue, we genetically fused MARCH5 ubiquitin ligase RING domain (residues 1–69) with either mitochondrial TOMM20 or peroxisomal PEX26 (residues 237–305) to generate organelle-specific targeting MARCH5, referred as mito-MARCH5(R) and po-MARCH5(R), respectively. The organelle-specific localization of mito-MARCH5(R) and po-MARCH5(R) was confirmed with immunofluorescence microscopy (Fig. 9 A). MARCH5 KO cells were transfected with either mito-MARCH5(R) or po-MARCH5(R), then treated with Torin1. In the presence of either WT MARCH5 or po-MARCH5(R), there was a significant reduction of peroxisome numbers upon Torin1 treatment (Fig. 9, A and B). In the presence of mito-MARCH5(R), peroxisome numbers did not change upon Torin1 treatment. In addition, folimycin blocked the reduction of peroxisome number in po-MARCH5(R)–expressing cells (Fig. 9 B). These results suggest that peroxisomal MARCH5 but not mitochondrial MARCH5 is responsible for Torin1-induced pexophagy.

We also investigated whether MARCH5 KO affects the downstream pexophagy pathway. Ubiquitinated peroxisome proteins recruit autophagic receptors p62 and NBR1 to mediate autophagosome formation (Deosaran et al., 2013; Kim et al., 2008). To exclude the possibility that MARCH5 affects the downstream pathway of PMP70 ubiquitination, we induced pexophagy by using the inducible FKBP (FK506 binding protein) and FRB (FKBP–rapamycin binding domain) heterodimerization system to mimic peroxisome ubiquitination. FRB(T2098L) was fused to PEX26 (residues 237–305) and coexpressed with cytosolic Ub-GFP-FKBP in HeLa cells (Fig. 9 C). In the presence of rapalog AP21967, a small molecule that induces heterodimerization of FRB and FKBP, Ub-GFP-FKBP was recruited to the peroxisome surface to mimic peroxisomal protein ubiquitination, which in turn induced pexophagy in WT HeLa cells (Fig. 9 D and Fig. S5 C). In MARCH5 KO HeLa cells, the addition of rapalog also significantly reduced peroxisome numbers (Fig. 9 E and Fig. S5 C), confirming that MARCH5 works upstream of peroxisome protein ubiquitination in pexophagy.

Altogether, we propose a new model for the function of MARCH5. MARCH5 can localize on peroxisomes via PEX19/PEX3 and plays a role in Torin1-induced pexophagy by mediating the ubiquitination of PMP70 (Fig. 9 F).

## Discussion

There are two main discoveries in this study. One is identifying MARCH5 as a dual-organelle localized protein that is also targeted to the peroxisome by PEX19. The other is that MARCH5 mediates PMP70 ubiquitination in Torin1-induced pexophagy. It is intriguing since MARCH5 was previously known as a mitochondrial ubiquitin ligase. Our findings suggest new molecular functions of MARCH5 on peroxisomes.

Other studies also indicated that MARCH5 may locate on peroxisomes. In a study addressing MARCH5 function on mitochondria, Koyano et al. (2019a) found that in HeLa cells overexpressing Parkin, the addition of mitochondria depolarization reagent carbonyl cyanide 3-chlorophenylhydrazone induced mitophagy and translocation of MARCH5 to peroxisomes. In another study addressing the role of MARCH5 in mitophagy, GFP was knocked in to fuse with the N-terminus of MARCH5 (Shiiba et al., 2021). GFP-MARCH5 expressing at the endogenous level displayed a separate population of punctate structures in addition to mitochondrial localization. These punctate structures were reminiscent of peroxisomes, similar to what we observed with overexpressed GFP-MARCH5. In the current study, we confirmed the peroxisomal localization of MARCH5 with co-immunofluorescence staining and fractionation experiments. Furthermore, we biochemically characterized PEX19 and PEX3 as the MARCH5-binding proteins that contribute to the peroxisomal localization of MARCH5.

A primary question is what is the function of MARCH5 on peroxisomes? Here, we show that MARCH5 mediates the ubiquitination of peroxisomal protein PMP70, which occurs during Torin1-induced pexophagy. In ubiquitin-dependent autophagy, proteins or organelles are modified with ubiquitin, then recognized by the autophagy adaptors to initiate the formation of autophagosomes. Although most studies of pexophagy in yeast indicate a ubiquitin-independent process (Farré et al., 2008; Motley et al., 2012a, 2012b), ubiquitination is sufficient to induce pexophagy in mammalian cells (Kim et al., 2008). PEX3 overexpression in mammalian cells induced ubiquitination-related pexophagy (Yamashita et al., 2014). Starvation with amino acid depletion induces pexophagy in a way dependent on ubiquitin ligase PEX2 and autophagy adaptor NBR1 (Sargent et al., 2016). Our results provide another example of ubiquitination-dependent pexophagy, suggesting a more general role for ubiquitination in mammalian pexophagy.

Various proteins, including PEX5, PEX3, and PMP70, are ubiquitinated during pexophagy (Nordgren et al., 2015; Sargent et al., 2016; Yamashita et al., 2014). Although we found that MARCH5 mediates ubiquitination of PMP70, we cannot exclude the possibility that other peroxisomal surface proteins are also ubiquitinated. We also found that PEX3 could be ubiquitinated by MARCH5 (Fig. S3 C). However, PEX3 ubiquitination and degradation seem to be paradoxical. Unlike PMP70, the PEX3 level did not decrease with Torin1 treatment (Fig. 5, C and D; and Fig. 7, A and B), but the addition of MG132 increased the PEX3 level in the Torin1-treated condition (Fig. 5 G). The level of PEX3 increased with MARCH5 knockdown, while other peroxisomal proteins remained unchanged (Fig. 4 G). It was reported that PEX3 is ubiquitinated and degraded in a proteasome-dependent manner on mitochondria when peroxisomes are depleted (Sugiura et al., 2017). It would be intriguing to see whether PEX3 relocates to mitochondria for proteasomal degradation in the Torin1-treated condition.

Our findings synergize well with several recent studies of MARCH5. MARCH5 and USP30 are a pair of ubiquitin ligase and deubiquitinase that reciprocally regulate protein ubiquitination on mitochondria. MARCH5 is associated with mitochondrial TOM complex to ubiquitinate proteins for elimination, while USP30 removes ubiquitin to promote the mitochondrial import of proteins (Phu et al., 2020). MARCH5 promotes mitophagy, while USP30 inhibits mitophagy (Bingol et al., 2014; Koyano et al., 2019b). Interestingly, USP30 also localizes on peroxisomes and has been identified to play an inhibitory role in pexophagy (Marcassa et al., 2018). Ablation of USP30 increases PEX5 and PMP70 ubiquitination and enhances pexophagy (Riccio et al., 2019). Since we found that the deletion of MARCH5 decreases PMP70 ubiquitination and inhibits pexophagy, it is possible that MARCH5 and USP30 also execute reciprocal regulation on peroxisomes.

MARCH5 might be regulated by mTOR signaling. Upon Torin1 treatment, there was a significantly increased ubiquitination level on peroxisomes in a MARCH5-dependent manner, but we do not know how this was induced. We have excluded the upregulation of MARCH5 expression (Fig. S5 D). MARCH5 may be redistributed between mitochondria and peroxisomes (Fig. S5 E). It is also likely that MARCH5 is activated via unknown posttranslational modifications. These areas are all worth exploring to further understand MARCH5 function and the molecular mechanism of pexophagy in the future.

## Materials and methods

### Antibodies

Detecting reagents, including streptavidin-HRP (1:2,000 for WB, 3999S), anti-Myc antibody (1:2,000 for WB, 2276S), anti-Myc HRP (1:1,000 for WB, 2040S), anti-HA antibody (1:2,000 for WB, 2367S), anti-HA HRP (1:1,000 for WB, 2999S), V5 (1:1,000 for immunofluorescence, 13202S), and anti-GFP HRP (1:1,000 for WB, 2037S) were purchased from Cell Signaling Technology. Antibodies against V5 (1:3,000 for WB, ab27671) and MARCH5 (1:2,000 for WB, ab174959) were purchased from Abcam. Anti-GDAPH (1:4,000 for WB, A00191) and anti–β-actin (1:4,000 for WB, A00702) were purchased from GenScript. Other antibodies used in this study included anti-FLAG (1:2,000 for WB, GNI14110-FG) and anti-FLAG HRP (GNI4310-FG; GNI), anti-PMP70 (1:3,000 for WB, SAB4200181; Sigma-Aldrich), and anti-GFP (1:3,000 for WB, M20004; Abmart). Anti–V5-HRP (1:5,000 for WB, R961-25) was purchased from Thermo Fisher Scientific. Other primary antibodies used were anti-MARCH5 (19168S; Cell Signaling Technology), anti-PEX19 (14713–1-AP; Proteintech), anti-PEX3 (sc-271477; Santa Cruz Biotechnology), anti-catalase (219010; Millipore), and anti-PEX13 (ab235043; Abcam). Secondary antibodies conjugated to HRP were purchased from GenScript.

### Molecular cloning

We mainly used pcDNA3.1 myc-His (+) B (V80020; Invitrogen) and pHR_EF1a (modified from pHR_PGK, #79120; Addgene) for transit transfection and virus packing, respectively. Human MARCH5 (including its variants), PEX3 (including its variants), PEX19 (including its variants), and PMP70 were cloned into pcDNA3.1 with a C-terminal Myc or V5 or triple FLAG tag using Gibson assembly. MARCH5-mCherry-Myc (including its variants) and PEX3-YFP (including its variants) were cloned into pcDNA3.1 and pHR_EF1a. mCherry-SKL, RFP-SKL, GFP-SKL, and GFP-RFP-SKL were cloned into pHR_EF1a. HA-Ub was purchased from Addgene (#18712). All plasmids were confirmed by Sanger sequencing.

### Cell culture and transfection

iPUP Jurkat-inducible cell line and PUP-IT^MARCH5^ cell line derived from iPUP were generated (Liu et al., 2018). Cells were cultured in RPMI 1640 supplemented with 5% FBS in 5% CO_2_ at 37°C. 293T cells (CRL-1573; ATCC) and HeLa cells (CCL-2; ATCC) were cultured in DMEM supplemented with 10% FBS in 5% CO_2_ at 37°C. All media and FBS were obtained from Life Technologies and Gemini. Transient transfections were performed using the Lipofectamine 3000 reagent (L3000015; Invitrogen) according to the manufacturer’s instructions. All the cell lines were also tested and confirmed negative for mycoplasma.

### Lentivirus generation, infection, and expression

GFP-SKL, RFP-SKL, MARCH5-mCherry-Myc, PEX3-YFP, and all their variants’ sequences were PCR amplified and cloned into a lentiviral vector (pHR_EF1a) by Gibson assembly using *BamH*I and *Not*I restriction sites. Lentiviral vectors and their respective packaging vectors (pCMV-dR8.91 and pMD2G) were cotransfected into 293T cells in a 10:10:1 molar ratio, respectively. Media were changed 16 h after transfection to low-volume media (6 ml per 10-cm dish). Media were collected at 48 h after transfection, replaced with fresh media (5 ml), and collected again at 72 h. Viral supernatant was cleared from cell debris via centrifugation (10 min at 1,000 *g*) as well as filtration through a 0.45-μm polyethersulfone membrane (SLHV033RB; Millipore).

### PUP-IT application, MS, and data analysis

PUP-IT assay was performed using PUP-IT^MARCH5^ stable cell line. Briefly, biotin (4 μM) and Dox (2 μg/ml) were added into PUP-IT^MARCH5^ and the other control cell line to start proximity labeling. After 48 h, cells were harvested and lysed by lysis buffer (50 mM Tris, 200 mM NaCl, 1% NP-40, pH 7.5). Then, lysate was treated by 8 M urea, 10 mM DTT at 56°C, and 25 mM iodoacetamide in the dark for 45 min. 50 μl streptavidin magnetic beads (88816; Pierce) were added into the lysate and shaken for 1 h at room temperature. The lysate was washed sequentially by buffer 1 (50 mM Tris, pH 8.0, 8 M urea, 200 mM NaCl, 0.2% SDS), buffer 2 (50 mM Tris, pH 8.0, 8 M urea), and buffer 3 (50 mM Tris, pH 8.0, 0.5 mM EDTA, 1 mM DTT). Finally, beads were resuspended with 100 mM ammonium carboxylate, and 6 μg trypsin (V5280; Promega) was added for on-bead digestion overnight at 37°C. The digested peptides were collected and cleaned with ZipTips (ZTC18S096; Millipore) before MS analysis.

Peptides were separated and analyzed on an Easy-nLC 1000 system coupled to a Q Exactive HF mass spectrometer (both from Thermo Fisher Scientific). Approximately 2 μg of peptides were separated in a homemade column (75 μm × 15 cm) packed with C18 AQ (5 μm, 300Å; Michrom Bioresources) at a flow rate of 300 nl/min. Mobile phase A (0.1% formic acid in 2% acetonitrile) and mobile phase B (0.1% formic acid in 98% acetonitrile) were used to establish a 60-min gradient comprising 2 min of 5% B, 40 min of 5%–30% B, 6 min of 30%–45% B, 2 min of 45%–90% B, and 10 min of 90% B. Peptides were then ionized by electrospray at 2.3 kV. A full MS spectrum (mass-to-charge ratio [m/z] range 375–1,400) was acquired at a resolution of 120,000 at m/z 200 and a maximum ion accumulation time of 20 ms. Dynamic exclusion was set to 30 s. Resolution for higher-energy C-trap dissociation (HCD) tandem MS (MS/MS) spectra was set to 30,000 at m/z 200. The automatic gain control setting of MS and MS2 were set at 3E6 and 1E5, respectively. The 20 most intense ions above a 1.7E4-count threshold were selected for fragmentation by HCD with a maximum ion accumulation time of 60 ms. Isolation width of 1.6 m/z units was used for MS2. Single and unassigned charged ions were excluded from MS/MS. For HCD, normalized collision energy was set to 25%.

The raw data were processed and searched with MaxQuant 1.5.4.1 with MS tolerance of 4.5 ppm and MS/MS tolerance of 20 ppm. The UniProt human protein database release 2016_07 (70,630 sequences; UniProtKB sequence A4QE80) and the database for proteomics contaminants from MaxQuant were used for database searches. Reversed database searches were used to evaluate the false discovery rate (FDR) of peptide and protein identifications. Two missed cleavage sites of trypsin were allowed. Oxidation (M), acetyl (protein N-terminus), deamidation (NQ), and GGE (K) were set as variable modifications. The FDR of both peptide identification and protein identification was set to be 1%. The option of second peptides, match between runs, and dependent peptides was enabled. LFQ was used to quantify the difference of protein abundances between different samples. The MS proteomics data have been deposited to the ProteomeXchange Consortium (http://proteomecentral.proteomexchange.org) via the iProX partner repository (Ma et al., 2018) with the dataset identifier IPX0003609000/PXD029348.

### Generation of KO cell lines by CRISPR/Cas9-based genome editing

HeLa cells lacking endogenous MARCH5 were generated by CRISPR/Cas9-mediated genome editing according to protocols published by the Zhang laboratory (Cong et al., 2013). In brief, we used an online CRISPR design tool (http://crispr.mit.edu) to select individual gRNA sequences targeting exons of genomic genes and designed the following oligonucleotides accordingly: MARCH5 sgRNA1 (5′-AGG​CAA​GAT​GAT​TCG​CTG​GGA​GG-3′), sgRNA2 (5′-ATT​AGG​CAA​GAT​GAT​TCG​CTG​GG-3′), and sgRNA3 (5′-TAT​TAG​GCA​AGA​TGA​TTC​GCT​GG-3′). Oligonucleotides were phosphorylated, annealed, and cloned into a modified pX330 (containing GFP) using the *Bbs*I restriction site. CRISPR constructs were first verified by sequencing and then transfected into cells. After 48 h, transfected cells were diluted and plated for single-cell clonal selections from a GFP-positive population. The GFP-positive cells were further screened for the absence of genes by immunoblotting (IB) using specific antibodies or by immunofluorescence imaging to verify the absence of peroxisomes.

### Immunofluorescence microscopy

Cells were plated on coverslips and maintained in 5% CO_2_ at 37°C for 24 h before staining. Cells were washed three times with 1× PBS and fixed in 4% PFA for 15 min, permeabilized in 1% NP-40 for 10 min, blocked with 2.5% BSA in PBS for 1 h at room temperature, and incubated with primary antibody overnight at 4°C. Secondary antibodies were applied for 1 h at 37°C, stained with DAPI for 2 min, and mounted using Mounting Medium (H-1000; Vector Laboratories). Confocal fluorescence imaging was performed using a Nikon Ti-E + A1Rsi confocal microscope with a 63×/1.4 NA Plan Apochromat Lambda oil objective and A1-DU4 4 Detector Unit (photomultiplier tube). Images were acquired at 16 bits by NIS-Elements Confocal software. For visual presentation, only the brightness was adjusted.

### PLA

Briefly, cells were fixed with 4% PFA for 10 min and washed three times with PBS. Cells were then lysed by 1% NP-40 in PBS for 10 min, followed by PBS three times, and incubated with blocking solution for 60 min at 37°C. Cells were incubated for 2 h with primary antibody as follows: anti-Myc (1:8,000; Cell Signaling Technology) and anti-V5 (1:1,000 Cell Signaling Technology). Duolink secondary antibodies were then added for 1 h at 37°C. Oligonucleotide-conjugated secondary antibodies were ligated together in a circle using the Duolink ligation solution for 30 min at 37°C, and polymerase was added to amplify the ligated circular oligonucleotides for 100 min at 37°C. Duolink red fluorescence was indicative of polymerized oligonucleotide signals. To label mitochondria or peroxisome, cells were infected by Mito-RFP (C10601; Invitrogen) or peroxisome-GFP (C10604; Invitrogen), respectively, before fixation. Signals were observed using a confocal microscope (Nikon Ti-E + A1Rsi).

### IP and WB assay

The cells were washed three times in PBS and lysed directly using cell lysis buffer (50 mM Tris, 200 mM NaCl, 1% NP-40, pH 7.5) for 1 h at 4°C. 100× protease inhibitor cocktail (B14001; Biomake) was added to the lysis buffer before use. Lysates were centrifuged at 4°C at maximum speed (15,000 *g*) for 10 min. The supernatant was subjected to BCA protein assay (DQ111-01; TransGen Biotech) to quantify protein levels. For IP, the cell lysates were incubated with the Myc/FLAG magnetic beads (M047-11/M185-11; MBL Life Sciences) for 1 h or overnight at 4°C. The beads were pelleted and washed with lysis buffer three times and heated in 1× denaturing loading buffer for 10 min at 95°C before being resolved by SDS-PAGE. The cell lysates were separated on a 4%–20% Bis-Tris gel (M00656; GenScript), transferred to polyvinylidene difluoride membranes (IPVH00010; Millipore), and probed with antibodies.

### In vivo ubiquitination assay

WT HeLa and/or MARCH5 KO cells were transfected with HA-Ub plasmids. After indicated treatment, cells were washed three times with PBS and pelleted in 1.5-ml Eppendorf tubes. Cell pellets were lysed with cell lysis buffer (20 mM Tris, 150 mM NaCl, 1% Triton X-100, pH 7.5) containing 0.5% SDS at 95°C for 10 min. Lysates were then diluted with cell lysis buffer to 0.1% SDS. 100× protease inhibitor cocktail (B14001; Biomake) was added to the lysis buffer before use. Lysates were centrifuged at 4°C at 15,000 *g* for 10 min. The cell lysates were then incubated with anti-PMP70 antibody for 2 h, then protein G beads (20398; Thermo Fisher Scientific) were added into the protein–antibody complex overnight at 4°C. The beads were washed three times with cell lysis buffer containing 500 mM NaCl and heated in 1× denaturing loading buffer for 10 min at 95°C before being resolved by SDS-PAGE.

### FACS analysis

HeLa cells stably expressing GFP-SKL or RFP-GFP-SKL were treated with reagents. Cells were washed three times with PBS and collected after digestion with trypsin. After washing three times with PBS, cells were resuspended in FACS buffer (1% BSA in PBS) and analyzed by CytoFLEX (Beckman Coulter). The data were analyzed using FlowJo version 10 software.

### Quantification and statistical analysis

Quantitative data are presented as mean ± SD. Error bars represent 1 SD from the mean. For comparisons of two samples, statistical significance was assessed using P values calculated by unpaired two-tailed Student’s *t* test in either GraphPad Prism or Perseus software. Multiple group comparisons were conducted using one-way ANOVA and Tukey’s multiple comparisons test. Comparisons of one control group to multiple other groups were performed using one-way ANOVA and Dunnett’s multiple comparisons test. Data distribution was assumed to be normal, but this was not formally tested. The statistical analyses were performed using GraphPad Prism. The number of experiments used for the statistical evaluation is specified in the figure legends.

### Online supplemental material

Fig. S1 shows that PEX19 peptides were identified in the proximity labeling experiments. Fig. S2 shows the effect of PEX19 knock down on MARCH5 location and pexophagy. Fig. S3 shows that PEX3 is ubiquitinated. Fig. S4 shows that Torin1-induced pexophagy also occurred in OVCAR8 cells. Fig. S5 includes the genome editing strategy to knock out MARCH5 in HeLa cells, representative images for main figures, and preliminary experiments for the discussion. Table S1 includes the source data for Fig. 1 D with protein names, intensity, and P values used for the plot in Fig. 1 D.

## Supplementary Material

Table S1 includes the source data for Fig. 1 D with protein names, intensity, and P values used for the plot in Fig. 1 D.
